# School-Based Homework Interventions for Improving 24-hour Movement Behaviours in Primary School Children: A Systematic Review and Meta-Analysis

**DOI:** 10.1186/s40798-025-00898-7

**Published:** 2025-08-09

**Authors:** April Forrest, Duncan Buchan, Nicholas Sculthorpe, Lawrence Hayes, Samantha Robinson

**Affiliations:** 1 School of Health and Life Sciences, University of the West of Scotland, Hamilton International Technology Park, Stephenson Place, Blantyre, Glasgow, G72 0LH UK; 2 Lancaster Medical School, Faculty of Health & Medicine, Sir John Fisher Driver, Lancaster University, Lancaster, LA1 4AT UK

## Abstract

**Background:**

School-based interventions aimed at improving physical activity (PA), sedentary behaviour (SB) and sleep (i.e., 24-hour movement behaviours) are prevalent. However, the potential use of homework as an intervention method has been largely unexamined. Our objective was to assess the effectiveness of school-based health interventions which implement homework to improve 24-hour movement behaviours in primary school-aged children, whilst examining the moderating effects of study characteristics on intervention effectiveness.

**Methods:**

We searched CINAHL, PubMed, Scopus, SPORTDiscus, The Cochrane Library and Web of Science on 4th March 2024 using the following eligibility criteria: (1) participants were aged 5–12 years old; (2) school-based interventions that implemented homework specifically designed to improve one or more 24-hour movement behaviours; (3) randomised- or non-randomised controlled trials, or mixed methods studies where quantitative components included experimental or quasi-experimental data that could be clearly extracted; (4) device-based measured changes in individual or combined 24-hour movement behaviours, or their compositions, were reported. Data were extracted independently by two reviewers with study quality rated using the NIH quality assessment tool. Random-effects meta-analyses were processed to compute standardised mean difference (Hedges’ g), with subgroup analyses, and meta-regressions also conducted.

**Results:**

From 2,281 studies, 19 studies involving 13,160 participants were included for data extraction. Meta-analyses revealed significant favourable association for school-based interventions which implemented homework for sleep outcomes (*g* = 1.06, *p* < 0.0001) and SB (*g* = -0.20, *p* = 0.0034). No significant effects of the interventions compared to controls were found for PA. Meta-regressions revealed that longer intervention durations significantly improved PA (counts per minute; β = 0.14, *p* = 0.0241), with no significant effects found for sleep or SB. Subgroup analyses showed significant effects of intervention on SB in RCT’s in both theory-based and non-theory-based studies, though differences between subgroups were not statistically significant. Effects varied between pre- and post-implementations of 24-hour movement guidelines on SB, but these differences were also not statistically significant.

**Conclusion:**

These results highlight a significant gap in school-based interventions implementing homework targeting all 24-hour movement behaviours, emphasising the need for future interventions to focus on reducing SB and improving sleep for more beneficial outcomes.

**Registration:**

PROSPERO CRD42024518271.

**Supplementary Information:**

The online version contains supplementary material available at 10.1186/s40798-025-00898-7.

## Key Points

There is a significant gap in school-based health interventions which implement homework and target all three 24-hour movement behaviours.

To the best of our knowledge, our systematic review and meta-analysis is the first to use device-based measured physical activity, sedentary behaviour and sleep when reviewing school-based health interventions, which implement homework, to improve 24-hour movement behaviours in children.

Even though current school-based interventions primarily focus on physical activity, our research suggest that focus should shift to concentrate more on reducing levels of sedentary behaviour and improving sleep; whilst examining the secondary effects on physical activity. This may lead to more substantial positive results in improving 24-hour movement behaviours, rather than continuing with the emphasis still placed on increasing physical activity.

**Supplementary Information:**

The online version contains supplementary material available at 10.1186/s40798-025-00898-7.

## Background

Historically, movement recommendations for children have heavily focused on physical activity (PA), particularly moderate-to-vigorous physical activity (MVPA), due to its well-established links to positive health outcomes and non-health benefits such as cognitive development and educational performance [1–3]. However, other movement behaviours, such as sleep and sedentary behaviour (SB), have received less attention in relation to overall health and well-being [4, 5]. Literature increasingly highlights the importance of achieving movement guidelines for children’s overall health, with positive associations to physical (e.g., adiposity) and psychosocial (e.g., well-being) outcomes, compared with meeting fewer or none of the guidelines [6]. While high levels of PA, low levels of SB and adequate sleep can be independently related to children’s health, it is important to view these behaviours collectively [7–9].

This shift in perspective, considering PA, SB, and sleep together, has led to the concept of 24-hour movement behaviours and an evolution in PA guidelines and research [10, 11]. Due to their compositional nature, PA, SB and sleep are intrinsically collinear with a change in one necessitating a compensatory adjustment in the others [12]. Acknowledging their interconnected nature, literature has moved away from the traditional paradigm of studying these behaviours in isolation and moved to a more holistic perspective, leading to the development of 24-hour movement guidelines [13]. These guidelines, for children and youth (5–17 years), were first published by Canada in 2016, followed by New Zealand and Australia in 2017 [11, 14, 15]. The guidelines stress the importance of higher levels of PA and sleep, and lower levels of SB for optimal health benefits [16]. Despite growing evidence, the United Kingdom (UK) is yet to produce or adopt 24-hour movement guidelines for children aged 5 to 12 years old.

Evidence suggests that few children meet all recommended movement behaviour guidelines. A recent review of PA, SB and sleep in the UK, revealed that only around 8% of children achieved all three of the Canadian 24-hour movement guidelines, while 11% met none [17, 18]. This outlines the discrepancy between the implementation of movement guidelines and the actual behaviours of children. Despite this, research examining adherence to 24-hour movement guidelines in the UK remains limited [18–20]. Further evidence from Canada and Australia suggests similar findings [21, 22]. Additionally, there is also an unfavourable shift in children’s movement behaviour as they transition from primary to secondary school, with increases in SB and decreases in sleep time and PA being observed [23]. Compliance during this transitional period with 24-hour movement guidelines drop significantly, highlighting the need for comprehensive interventions [23]. Other research outlines the role of familial influence in promoting healthy behaviours [24]. The home environment exerts significant influence on children’s activity levels with parents providing the opportunity, means and support for their children to be active [25]. With children spending around half of their day in the home setting, only a limited proportion of this time is spent active [26, 27]. Tandon et al., [27] reported that around 50% of children’s overall sedentary time and MVPA, respectively, was accrued at home.

In contrast, children spend the remainder of their waking hours in the school setting, presenting a convenient environment for health promotion activities [28]. Schools offer a strategic intervention setting due to their diverse demographic, irrespective of ethnicity, gender and socio-economic status [29]. Identifying variations in adherence to movement guidelines, a systematic review highlighted lower compliance with increased child age, lower adherence in girls, and reduced adherence in countries with lower Human Development Indices [17]. In addition to these demographic differences, intervention effectiveness can be further influenced by moderating factors such as parental involvement and use of theoretical frameworks. For example, one review outlined that direct parental engagement has the potential to improve children’s PA and SB [30]. Another review supported this but also highlighted that school-based interventions tend to be more effective at improving PA when underpinned by behaviour change theories during the design phase [31]. With their broad reach, schools not only have the potential to address these disparities but also offer a unique opportunity to engage parents and guardians in health promotion activities. In doing so they create a crucial bridge between school and home environments.

One approach of linking school-based interventions to the home environment is homework, which can provide a structured extension of school-based activities [32]. It also facilitates collaboration between parents and school staff, ensuring a consistent health promotion environment for children while reinforcing movement related behaviour change beyond school hours [33–35]. This strategic integration of homework into school-based health interventions recognises the potential for sustained engagement with children’s movement behaviours, leveraging the collaborative efforts of parents and educators.

Despite the literature indicating a promising potential of schools and the use of homework as focal points for children’s interventions, there is limited evidence in achieving sustained improvements in 24-hour movement behaviours. Effectiveness within this review refers to these interventions’ ability to produce long-term changes in children’s PA, SB, and sleep. Therefore, there is a need for a comprehensive review of school-based health interventions that implement homework to establish their impact on 24-hour movement behaviours.

Despite the potential benefits of integrating homework into school-based interventions, the current body of evidence highlights a notable absence in comprehensive reviews focusing on all three 24-hour movement behaviours. Therefore, the aim of this study is to: (1) assess the effectiveness of school-based health interventions which implement homework to improve 24-hour movement behaviours in primary school-aged children, and (2) examine the moderating effects of study characteristics (e.g., theory, study design, publication of 24-hour movement guidelines, time of day, accelerometer placement) on intervention effectiveness.

## Methods

The review adhered to The Preferred Reporting Items for Systematic Reviews and Meta-Analyses (PRISMA) guidelines checklist (supplementary file 1) [36] and was registered with the International Prospective Register of Systematic reviews (registration number CRD4202451827).

### Information Sources and Search Strategy

Electronic databases (*n* = 6) were searched from inception to Monday 4th March 2024 for peer-reviewed journal articles published in English, with no limits on publication dates. These databases included CINAHL (EBSCO), PubMed, Scopus, SPORTDiscuss (EBSCO), The Cochrane Library and Web of Science (core collection). A search strategy was developed using the Population, Intervention/Exposure, Comparison, Outcome and Study Design (PICOS) framework, endorsed by the Cochrane Collaboration [37]. Due to the importance of a homework component within this review, the full-text search field was used with ‘homework’ to ensure it was not missed when searching. The full search strategy for each database can be found in supplementary file 2. The reference lists of all included articles and relevant reviews were also screened for additional articles.

### Eligibility Criteria

The inclusion criteria for the study was informed by the PICOS eligibility criteria [38]. For the purpose of this review, we defined homework as all “tasks assigned to students by schoolteachers that are meant to be carried out during non-school hours” [39]. To be eligible, the homework component had to be home-based, however it did not need to be the primary method of intervention. The specific delivery methods or level of parental involvement were not considered as exclusion criteria.

Studies with the following characteristics were included in our review: (1) participants were aged 5–12 years old; ( (2) school-based interventions that implemented homework specifically designed to improve one or more 24-hour movement behaviours (i.e., PA, SB, and/or sleep); (3) randomised controlled trial (RCT) or non-randomised controlled trial (non-RCT), or mixed methods studies where the quantitative components included experimental or quasi-experimental data that could be clearly extracted; (4) they reported data on device-based measured changes in individual or combined 24-hour movement behaviours (PA, SB, and sleep), or their compositions (e.g., MVPA, light physical activity (LPA), moderate physical activity (MPA), vigorous physical activity (VPA), overall sedentary time, total sleep time, sleep efficacy, sleep onset latency and wake after sleep onset), measured via accelerometery (e.g., ActiGraph, activPAL, GENEActiv) [40].

Studies with the following characteristics were excluded from the review: (1) studies that were prescriptive to a specific target audience such as those with either physical disabilities, intellectual disabilities, endocrine disorders, or chronic diseases; (2) all grey literature; (3) studies that did not report device-based measured 24-hour movement behaviours (PA, SB or sleep).

Accelerometers were selected as the preferred measurement tool due to their ability to capture movement intensity, duration, and patterns of PA, SB and sleep over a 24-hour period [41], which were key outcomes of this review. Due to the need for comprehensive measurement of movement behaviours across the full 24-hour period, pedometers, which only capture step count and do not assess movement intensity or duration [42, 43], were excluded.

### Selection Process and Data Extraction

From the search all identified article citations were imported into EndNote 21 software (Thomson Reuters, San Francisco, CA, USA). Duplications were then removed before the remaining articles titles and abstracts were exported to the Rayyan application for further duplication removal and then screening [44]. Titles and abstracts were then independently screened by two reviewers (A.F. and S.D.) to determine if they met the criteria for inclusion in the review. The selected articles included by reviewers were then screened based on their full text for eligibility against the inclusion criteria. Any uncertainty on whether to include or exclude a specific article was discussed between the two reviewers, with a third reviewer (N.S.) informed of any discrepancies in inclusion. The third reviewer then held the casting vote to reach agreement.

Data extraction was performed by two reviewers (A.F., and S.D.) using an Excel spreadsheet (Microsoft^®^ Excel 2016, Microsoft Corporation, Redmond, WA, USA) informed by the PICOS criteria. This included population characteristics (e.g., sample size and age), intervention description (e.g., duration, element of homework), study design (e.g., RCT and non-RCT), outcome characteristics (e.g., movement behaviour and measurement method) and results data (e.g., study means, standard deviation (SD)). In studies where data was missing or poorly reported, corresponding authors were contacted by email. The data requested included sample size (*N*), mean, and SD of the movement behaviour at baseline and at follow-up for both intervention and control groups. This follows recommendations from The Cochrane Collaboration [45].

### Statistical Analysis

#### Narrative Synthesis of Data

Each behaviour construct was analysed in connection to homework, assessing its role as an intervention method and evaluating the overarching outcome of the intervention. Interventions that reported a favourable impact on movement behaviours were outlined as positive outcomes, with results reporting adverse impacts deemed as negative outcomes. This allowed for a clear distinction during the analysis stage.

#### Meta-analysis of Data

To assess the overall and differential intervention effects on PA, SB and sleep, mean change scores and SD from baseline to follow-up were calculated for both control and intervention groups. Where studies had multiple follow up periods the post-intervention follow-up time closest to the end point of the intervention was used for analysis. Meta-analyses were conducted in R (version 4.2.1; R Group for Statistical Computing) using the meta, metafor, and metareg packages [54]. To align the scoring with the direction of an improvement, certain variables (i.e., sleep latency) were reverse scored, so a positive effect size in the results always indicated a favourable effect of intervention. Furthermore, to ensure consistency and precision in reporting, data was converted from hours/day to mins/day before conducting calculations where required. Studies were assigned ‘HIP’ or ‘WRIST’ worn regarding device placement based on either the study reporting the placement or through inference by authors (D.B. and A.F.) via the cut-points used.

From the included studies, effect sizes were determined using Hedges’ *g* (standardised mean differences (SMD)) reporting 95% confidence intervals (CI) for the difference between arms (i.e., intervention vs. control) for each of the three behaviour outcomes, and for the derivatives of PA. A random-effects model (DerSimonian-Laird) was used to calculate pooled estimates to account for the likely variation in study design, methodology, populations, and outcome measurements. Heterogeneity between studies was quantified with the I^2^ statistic and assessed visually using forest plots. An I^2^ value of 25% was interpreted as low, 50% as moderate, and 75% as high heterogeneity [46]. Publication bias was assessed visually using funnel plots, with asymmetry indicating potential publication bias [47]. Egger’s Test was performed on asymmetrical funnel plots to determine if their asymmetry is significant (*p* < 0.05) which may suggest the presence of publication bias [48]. This was conducted where > 10 studies were included in a meta-analysis, which provided sufficient power to detect real asymmetry [37].

Pre-planned meta-regressions looking at duration were performed using a mixed-effects model to determine if duration explained the heterogeneity in effect sizes. Subgroup analyses were conducted to examine whether magnitudes of effect were dependent upon study characteristics of interest (i.e., theory, study design, publication of 24-hour movement guidelines, time of day, accelerometer placement). The statistical significance threshold was set at *p* ≤ 0.05.

### Risk of bias

The methodological quality of each study was independently assessed by two reviewers (A.F. and S.D.) using The Quality Assessment Tool for Controlled Intervention Studies [49] (see supplementary file 3 for additional information). A final quality assessment score was afforded to each study by the two reviewers (A.F. and S.D.) after thorough review of characteristics that constitute study quality following guidance from the National Health Institute (NIH) [49]. The scores were as follows: scores of thirteen and fourteen were deemed good; those scoring eight and lower were considered poor; and those that fell between nine and twelve were represented as fair. Any disagreements in judgement or justification for judgements were discussed between reviewers until an agreement was reached. All disputes (*n* = 6) were resolved and consultation from a third reviewer was not required. This assessment was used to measure the strength of the scientific evidence of the included studies and was not used to determine the inclusion of studies [50].

## Results

### Study Selection

**Fig. 1:**
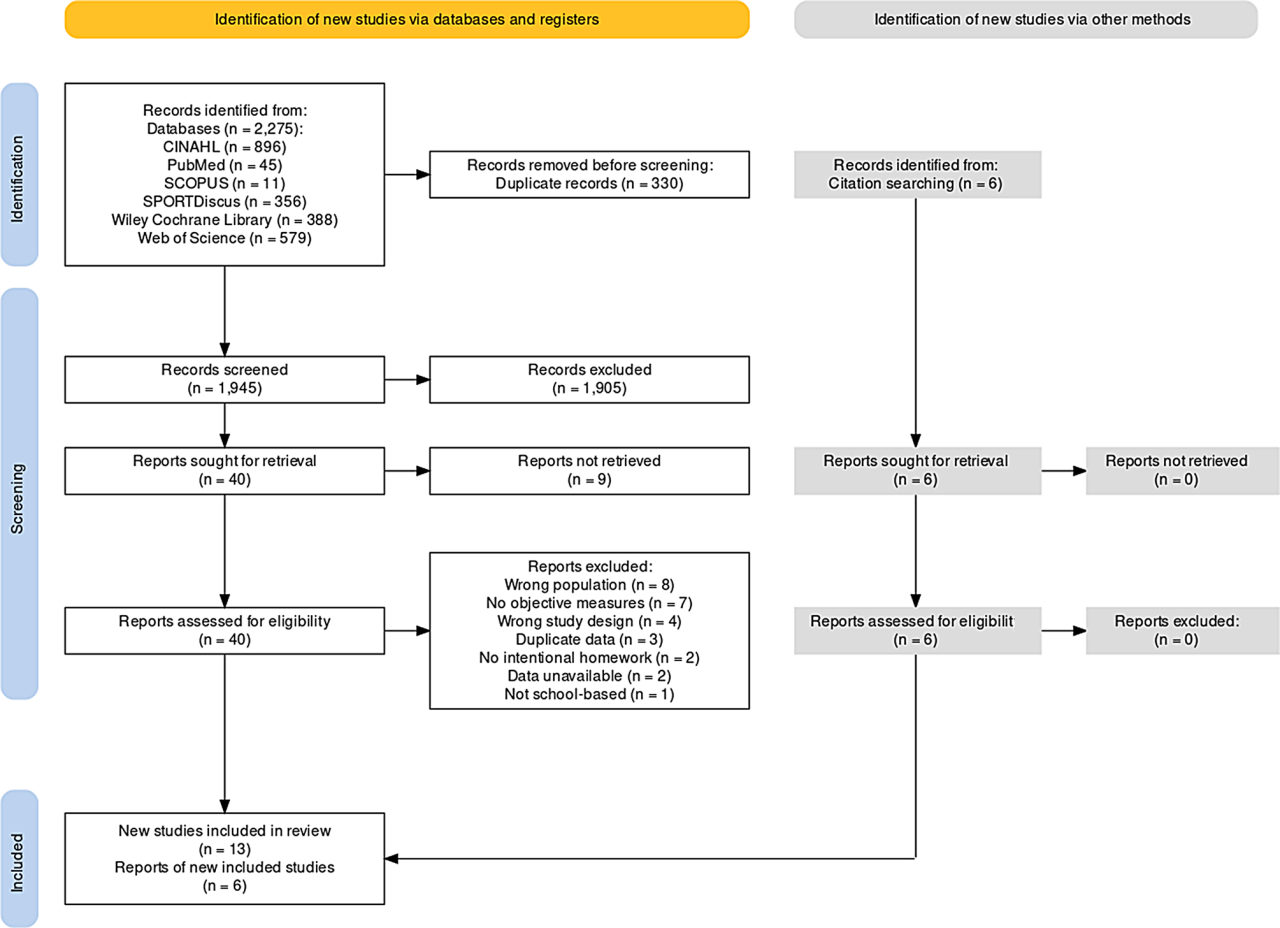
PRISMA Flow diagram

A total of 2,281 studies were initially identified, with 1,951 being screened after removing duplicates (*n* = 330) via EndNote Reference Manager and Rayyan software. Of the 46 articles screened by the full text, 27 were excluded [51–77], and 19 studies which met the eligibility criteria were included [78–96]. A PRISMA flowchart illustrating the study selection process can be found in Fig. 1 (see supplementary file 4 for additional information).

### Characteristics of Studies Included

Characteristics of the 19 studies are shown in Table 1, being published between 2005 and 2023. All studies included in the review used accelerometery to assess movement behaviours. The devices used included ActiGraph models [78–83, 85–88, 90–95], activPAL [82], CSA Model Activity Watch [96], ActiWatch [84], and GeneActiv [89]. The sample size varied from 71 [82, 84] to 2,221 [79, 86] participants with mean age varying between 8.3 [92] and 10.9 [95] years, all within the eligible age range of 5–12 years. The proportion of females varied from 40 to 61% [85, 95]. The duration of interventions was wide-ranging from 1.5 months [84, 95] to 30 months [92]. Five studies were conducted in England [79, 83, 86, 89, 94] and three in Norway [78, 91, 93], with the rest from Australia (*n* = 2) [81, 92], Switzerland (*n* = 2) [88, 90], America (*n* = 1) [96], Belgium (*n* = 1) [95], Canada (*n* = 1) [84], China (*n* = 1) [85], Netherlands (*n* = 1) [87], Northern Ireland (*n* = 1) [80], and Scotland (*n* = 1) [82].

**Table 1:** Study characteristics

Author (year)	Country	Study characteristics; Age group (mean age); Sex (M/F %)	Total number of participants		Intervention; Treatment Arms Behaviour (construct); Duration	Comparator	Accelerometer; Device Placement	Quality assessment score
			Intervention	Control				
Aadland et al. [78] (2019)	Norway	Design: Cluster RCT Age group: 10 years (10.2) Sex: 52.1/47.9	*n* = 1,129		Arms: 1 Behaviour: PA (TPA & MVPA) & SB Duration: 7 months	Control: No intervention.	Device: ActiGraph GT3X+ Placement: Right hip.	Fair (10)
			596	533				
Anderson et al. [79] (2016)	England	Design: Cluster RCT Age group: 8–11 years (9.5) Sex: 49.5/50.5	*n* = 2,221		Arms: 1 Behaviour: PA (MVPA) & SB Duration: 12 months	Control: No intervention.	Device: ActiGraph GT3X+ Placement: Hip*.	Good (12)
			1064	1157				
Breslin et al. [80] (2012)	Northern Ireland	Design: Non-RCT Age group: 8–9 years (9.11) Sex: 48.3/51.7	*n* = 416		Arms: 1 Behaviour: PA (LPA, MPA, VPA, MVPA) & SB. Duration: 3 months	Control: No intervention.	Device: ActiGraph GT3X Placement: Hip*.	Fair (9)
			209	207				
Cohen et al. [81] (2015)	Australia	Design: Cluster RCT Age group: 7–10 years (8.5) Sex: 45.9/54.1	*n* = 460		Arms: 1 Behaviour: PA (TPA & MVPA) Duration: 12 months	Control: No intervention.	Device: ActiGraph GT3X+ Placement: Hip*.	Fair (11)
			199	261				
Donnelly et al. [82] (2024)	Scotland	Design: RCT Age group: 9–12 years (10.3) Sex: 45.1/54.9	*n* = 71		Arms: 1 Behaviour: PA (MVPA), SB & Sleep Duration: 2 months	Control: No intervention.	Device: ActiGraph GT3X+ & activPAL4 Placement: Wrist and right thigh, respectively.	Fair (9)
			40	31				
Fairclough et al. [83] (2013)	England	Design: Cluster RCT Age group: 10–11 years (10.65) Sex: NR.	*n* = 316		Arms: 1 Behaviour: PA (LPA, MPA, VPA) & SB. Duration: 5 months	Control: No intervention.	Device: ActiGraph GT1M Placement: Hip*.	Poor (8)
			166	152				
Goran & Reynolds., [96] (2005)	America	Design: Cluster RCT Age group: 8–10 years (9.4) Sex: 41/59	*n* = 122		Arms: 1 Behaviour: PA (TPA). Duration: 2 months	Control: CD-ROM not related to health outcomes.	Device: CSA model 7164 activity monitor Placement: Right hip.	Poor (8)
			63	59				
Gruber et al. [84] (2016)	Canada	Design: Non-RCT Age group: 7–11 years (8.45) Sex: 43.7/56.3	*n* = 71		Arms: 1 Behaviour: sleep Duration: 1.5 months	Control: No intervention.	Device: ActiWatch-64 Placement: Non-dominant wrist.	Poor (6)
			46	25				
Ha et al. [85] (2021)	China	Design: RCT Age group: NR (10.0) Sex: 60/40	*n* = 171		Arms: 1 Behaviour: PA (MVPA) Duration: 6 months	Control: No intervention	Device: ActiGraph wGT3X-BT Placement: Waist.	Fair (10)
			85	86				
Kipping et al. [86] (2014)	England	Design: Cluster RCT Age group: 9–10 years (9.5) Sex: 49.2/50.8	*n* = 2,221		Arms: 1 Behaviour: PA (MVPA) & SB Duration: 8 months	Control: No intervention.	Device: ActiGraph GT3X+ Placement: Hip*.	Good (12)
			1064	1157				
Kocken et al. [87] (2016)	Netherlands	Design: Cluster RCT Age group: 9–11 years (9.16) Sex: 48.3/51.7	*n* = 1112		Arms: 1 Behaviour: PA (MVPA, TPA) & SB. Duration: 8 months	Control: No intervention	Device: ActiGraph Placement: Right hip.	Poor (8)
			615	497				
Kriemler et al. [88] (2010)	Switzerland	Design: Cluster RCT Age group: 6–10 years (9.3) Sex: 48.8/51.1	*n* = 502		Arms: 1 Behaviour: PA (MVPA, TPA) Duration: 9 months	Control: No intervention	Device: ActiGraph, MTI/CSA 7164/GT1M Placement: Hip.	Fair (10)
			297	205				
Lloyd et al. [89] (2018)	England	Design: Cluster RCT Age group: 9–10 years (9.75) Sex: 48.7/51.3	*n* = 1324		Arms: 1 Behaviour: PA (TPA, LPA, MPA, MVPA, VPA) & SB Duration: 12 months	Control: No intervention	Device: GENEActiv Placement: Non-dominant wrist.	Good (12)
			676	648				
Meyer et al. [90] (2014)	Switzerland	Design: Cluster RCT Age group: 6–10 years (9.3) Sex: 48.8/51.2	*n* = 502		Arms: 1 Behaviour: PA (TPA, MVPA) Duration: 9 months	Control: No intervention	Device: MTI/CSA 7164/GT1M, ActiGraph Placement: Hip.	Fair (10)
			297	205				
Resaland et al. [91] (2016)	Norway	Design: Cluster RCT Age group: 10 years (10.2) Sex: 52.1/47.9	*n* = 1129		Arms: 1 Behaviour: PA (TPA, MVPA, LPA) & SB Duration: 7 months	Control: No intervention	Device: ActiGraph GT3X+ Placement: Right hip.	Poor (7)
			596	533				
Salmon et al. [92] (2023)	Australia	Design: Cluster RCT Age group: 8–9 years (8.3) Sex: 42.7/57.3	*n* = 342		Arms: 3 Behaviour: PA (MVPA) & SB Duration: 30 months	Control: No intervention	Device: ActiGraph GT3X+ Placement: Right hip.	Fair (9)
			253	89				
Seljebotn et al. [93] (2019)	Norway	Design: Cluster RCT Age group: 9–10 years (NR) Sex: 51/49	*n* = 447		Arms: 1 Behaviour: PA (MVPA, LPA) & SB. Duration: 10 months	Control: No intervention	Device: ActiGraph GT1M/ GT3X/ GT3X+ Placement: Right hip.	Poor (8)
			228	219				
Taylor et al. [94] (2018)	England	Design: Cluster RCT Age group: 9–10 years (NR) Sex: 49.1/50.9	*n* = 232		Arms: 1 Behaviour: PA (MVPA, LPA, TPA) & SB Duration: 2 months	Control: No intervention	Device: ActiGraph GT9X Placement: Hip*.	Fair (9)
			117	115				
Verloigne et al. [95] (2012)	Belgium	Design: Cluster RCT Age group: 10–12 years (10.9) Sex: 39/61	*n* = 372		Arms: 1 Behaviour: PA (MVPA) & SB Duration: 1.5 months	Control: No intervention	Device: ActiGraph accelerometer Placement: Right hip.	Poor (6)
			141	231				

Abbreviations: RCT, randomised controlled trial; PA, physical activity; SB, sedentary behaviour; TPA, total physical activity; MVPA, moderate-to-vigorous physical activity; LPA, light physical activity; MPA, moderate physical activity; VPA, vigorous physical activity; n, number of participants; NR; not reported; M, male; F, female

Note: *Inference of device placement

### Movement Behaviour Composition

Almost two-thirds of the included studies investigated both PA and SB (*n* = 12) [78–80, 83, 86, 87, 89, 91–95]. Whereas 5 studies [81, 85, 88, 90, 96] investigated PA only and one study investigated just sleep [84]. There was only one study that investigated PA, SB and sleep together [82]. Specific PA behaviour constructs examined in the studies included MVPA, LPA, VPA, MPA, stepping time, and total physical activity (TPA). SB constructs included sitting time, standing time, sitting for 60 min plus, and inactivity. Finally, sleep behaviour constructs included total sleep duration, true sleep (the amount of time between sleep start and sleep end according to accelerometery data), sleep efficacy (the time in bed spent sleeping) and sleep latency (the amount of time taken from bedtime until the participant falls asleep) [97].

### Intervention Effectiveness

Characteristics of the interventions are shown in Table 2 including intervention information and specific homework components. Additionally, the effectiveness of each intervention on their primary outcomes is delineated as either having a positive (+), negative (-), or neutral impact (=). Studies which outlined a positive impact in one or more of its outcomes, but with no impacts to other primary outcomes, are represented as (+/=). Although most studies (*n* = 16) [79–88, 90, 92–96] outlined PA, SB or sleep as a primary outcome, they were examined alongside other primary outcomes such as fundamental movement skills (FMS), academic performance, or body mass index (BMI). Only three studies did not report either of the 24-hour movement behaviours as a primary outcome of their intervention [78, 89, 91].

Interventions with a positive impact on their primary outcomes (*n* = 6) [81, 83, 84, 88, 92, 93] reported increases in MVPA [81, 88, 93], decreases in SB [83, 92], or improvements in sleep [84]. Those which reported a neutral impact on primary outcomes (*n* = 8) [78, 79, 85–87, 90, 91, 95] reported no significant change in PA [78, 79, 91], no increased MVPA [85, 87], a decrease in MVPA [86], and one study reported that initial effects of PA were not sustained after three years [90]. Those which reported mixed results on their primary outcomes (*n* = 4) [80, 82, 94, 96] also reported mixed outcomes on movement behaviour. This included significant observed effects for VPA, MPA and LPA [80], increased sleep duration [82], non-significant differences of PA [82, 96], and a reduction in SB during the school day [94].

Only one study reported a negative impact of intervention on their primary outcome of preventing excessing weight gain with BMI increasing throughout the intervention [89]. Lloyd et al., [89] also reported that there were no significant differences in PA or SB from baseline to follow-up at 18 months.

(**Insert** Table 2: Intervention Characteristics.)

**Table 2:** Intervention characteristics

Author (year)	Intervention; Name Content Primary outcome(s)	Theory; Model	Homework content	Intervention effect^1^ + / = / -	Behaviour outcome findings
Aadland et al. [78] (2019)	Active Smarter Kids (ASK) Physical Activity (PA) Intervention. **Content**: (1) PA educational lessons, (2) PA breaks, (3) PA homework. In all adding 165 min of PA to the mandatory 135 min of PA and physical education. **Primary outcome**: Increase FMS.	NR.	PA homework with children receiving a tennis ball and skipping rope.	=	There were no significant differences between groups for change in PA during school hours (*p* ≥ 0.303) or during the whole day (*p* ≥ 0.440).
Anderson et al. [79] (2016)	Active for Life Year 5 (AFLY5). **Content**: (1) Training for classroom teachers and learning support assistants, (2) provision on 16 lesson plans and teaching materials for teachers/ learning assistants to deliver, (3) provision of 10 parent-child interaction homework activities, (4) information for schools to include in school newsletters, (5) information for parents on how to encourage their children to eat healthily and be active. **Primary outcomes**: improve PA and diet.	SCT.	10 parent-child interaction homework activities. Included activities like ‘Freeze my TV” in which a specific time that would normally be spent watching television would be replaced with physically active play involving the parents and other family members that the child would write a log about.	=	PA or SB did not differ between children in schools allocated to the intervention, compared with those in control schools at the end of the long-term follow-up (1-year post-intervention).
Breslin et al. [80] (2012)	Sport for LIFE (living, integration, fun and education). **Content**: (1) Weekly 1 h activity sessions delivered to children on health, fun games and nutrition, (2) end point physical activity festival with Olympian in attendance, (3) teachers resource pack with 12 week-by-week teaching resources, (4) educational DVD and website which outlined the activities and sessions. **Primary outcomes**: increase PA, decrease SB, reduce screen time, reduce BMI, encourage healthy behaviour to nutrition.	SCT.	PA homework sessions which were provided via website and information pack to teachers.	=/+	Significant effects were observed for VPA, MPA, and LPA for the intervention group at follow-up. SB was significantly reduced for the intervention group but not for the control group.
Cohen et al. [81] (2015)	SCORES intervention. **Content**: (1) Teacher professional learning, student leadership workshops and PA promotional tasks, (2) implementation of PA policies in school to support PA and FMS within schools and home environment, (3) addressed strategies to improve school-community links. **Primary outcomes**: increase PA and improve FMS.	Socioecological model.	FMS homework to engage parents and encourage them to support children’s PA.	+	There was a statistically significant group–time interaction in favour of the intervention group for daily MVPA minutes (*P* = 0.008), corresponding to a difference of 13 min/day of MVPA. There was also a statistically significant group–time interaction in favour of the intervention group for daily after-school MVPA minutes (*P* = 0.028) and daily weekend MVPA minutes (*P* = 0.034). The changes in total PA (*P* = 0.054), MVPA percentage (*P* = 0.051), and within-school MVPA (*P* = 0.182) from baseline to post-test showed trends in favour of the intervention group. Overall, the intervention maintained daily MVPA.
Donnelly et al. [82] (2024)	Happy Homework (HH). **Content**: (1) Teachers provided with 8 weeks of HH activities and asked to deliver 6 of the activities, (2) HH workbooks given to children 3 x weekly to encourage involvement in activities. **Primary outcomes**: improve PA, limit SB, improve sleep and encourage positive dietary behaviours.	SDT.	Homework activities which aimed to improve both activity-related behaviours across the whole day and key dietary behaviours.	=/+	Post intervention stepping time and sleep duration were significantly greater for the intervention group in comparison to the control group. There were no significant differences post-intervention between the PA of the intervention and control groups.
Fairclough et al. [83] (2013)	Children’s Health, Activity and Nutrition: Get Educated! (CHANGE!) Project **Content**: Consisted of (1) teacher-led curriculum, (2) learning resources, and (3) homework tasks. **Primary outcomes**: increase PA, improve food intake and body size.	SCT.	Homework supplemented classroom work and targeted family involvement in food and PA related tasks.	+	At follow-up there was a significant intervention effect for LPA (β = 25.97 (95% CI = 8.04, 43.89) min, *p* = 0.01). No intervention effects were observed for MPA and VPA. At follow-up non-significant between group differences were observed for sedentary time (β=−8.44 (95% CI = − 53.23, 36.35) min).
Goran & Reynolds [96] (2005)	Interactive Multimedia for Promoting Physical Activity (IMPACT) CD-ROM **Content**: Intervention involved a (1) interactive computer game (CD-ROM), supplemented by (2) classroom assignments and (3) homework. **Primary outcomes**: improving PA and reducing obesity.	SCT.	Homework supplemented the CD-ROM interactive multimedia.	=/+	There were no significant effects on total PA by accelerometery. There was an overall treatment effect on the reduction of time spent in MPA, and changes in time spent in LPA (reducing in boys and increase in girls).
Gruber et al. [84] (2016)	Sleep For Success (SFS) **Content**: The programme included four modules, (1) sleep knowledge and education, (2) Family and community involvement, (3) sleep promotion for staff, (4) Sleep-friendly school environment. Six classes offered during school time to students. **Primary outcomes**: improve sleep and academic performance.	NR.	Homework activities not outlined further.	+	Intervention group true sleep^2^ was increased by 18.2 min/night, sleep efficiency by 2.3% and sleep latency was shortened by 2.3 min.
Ha et al. [85] (2021)	Active 1 + Fun **Content**: Consisted of (1) ten 30- minutes workshops followed by 60-minutes activity classes, with one or more parent attending the session together with the child. **Primary outcomes**: increase MVPA, and parent-child co-activity.	SDT.	Weekly parent-child PA homework that encouraged children to spend at least 30 min in total per week to engage in parent-child PA.	=	No significant intervention effects were found for children’s and parents’ accelerometer measured MVPA. There was an increase from time point 1 to 2 in MVPA, but it remained the same between time point 1 and 3. The intervention was not effective at improving children’s MVPA.
Kipping et al. [86] (2014)	Active for Life Year 5 (AFLY5). **Content**: (1) Training for classroom teachers and learning support assistants, (2) provision on 16 lesson plans and teaching materials for teachers/ learning assistants to deliver, (3) provision of 10 parent-child interaction homework activities, (4) information for schools to include in school newsletters, (5) information for parents on how to encourage their children to eat healthily and be active. **Primary outcomes**: improve PA, reduce SB, and increase fruit and vegetable consumption.	SCT.	10 parent-child interaction homework activities. Included activities like ‘Freeze my TV” in which a specific time that would normally be spent watching television would be replaced with physically active play involving the parents and other family members that the child would write a log about.	=	The difference in means comparing the intervention group with the control group was − 1.35 (95% confidence interval − 5.29 to 2.59) mins/day for MVPA, and − 0.11 (–9.71 to 9.49) mins/day for SB.
Kocken et al. [87] (2016)	Extra Fit! (EF!) **Content**: A variety of (1) theory and practical lessons on PA and nutrition to reduce and prevent overweightness in primary school children, through (2) knowledge, attitude, social norm, and perceived behavioural control changes. **Primary outcomes**: improve dietary habits, physical activity and inactivity, to prevent overweightness.	Theory of planned behaviour.	PA homework which involved the parents in the homework.	=	Mean daily PA level and time spent on MVPA, did not differ significantly between the intervention group and control group. A trend effect (*p* < 0.10) could be observed for mean time spent inactive. Inactivity increased more in the control group than in the intervention group between T0 and T2.
Kriemler et al. [88] (2010)	KISS **Content**: Multi-component physical activity programme that included (1) structuring the three existing physical education lessons each week and (2) adding two additional lessons a week, (3) daily short activity breaks, and (4) physical activity homework. **Primary outcomes**: increase aerobic fitness, PA, and quality of life whilst decreasing body fat and composite cardiovascular risk factor scores.	Socioecological model.	Children received daily PA homework of about 10 minutes’ duration prepared by the PE teachers. This included aerobic, strength, or motor skill tasks such as brushing their teeth while standing on one leg, hopping up and down the stairs, rope jumping, or comparable activities.	+	Z scores increased more favourably for MVPA in school (1.19, 0.78 to 1.60; *P* < 0.001), all day MVPA (0.44, 0.05 to 0.82; *P* = 0.03), and TPA in school (0.92, 0.35 to 1.50; *P* = 0.003). Z scores for overall daily PA (0.21, − 0.21 to 0.63) did not change significantly. The intervention improved PA.
Lloyd et al. [89] (2018)	Healthy Lifestyles Programme (HeLP) **Content**: Included dynamic and interactive activities such as (1) physical activity workshops, (2) education sessions delivered by teachers with short homework tasks, (3) drama sessions, and (4) setting goals to modify behaviour. **Primary outcomes**: prevent excessive weight gain.	NR	Short and simple homework tasks were given at the end of each session for the children to complete in time for their next session.	-	No significant differences in any PA or SB from baseline to follow up at 18-months.
Meyer et al. [90] (2014)	KISS **Content**: Multi-component physical activity programme that included (1) structuring the three existing physical education lessons each week and (2) adding two additional lessons a week, (3) daily short activity breaks, and (4) physical activity homework. **Primary outcomes**: increase aerobic fitness, PA, and quality of life whilst decreasing body fat and composite cardiovascular risk factor scores.	Socioecological model.	Daily PA homework of about 10 min.	=	After initial beneficial effects in PA were reached by a multi-component physical activity intervention in school over an academic year, sustained benefits after three years were not seen.
Resaland et al. [91] (2016)	Active Smarter Kids (ASK) **Content**: (1) 90 min/week of physically active educational lessons mainly carried out in the school playground; (2) 5 min/day of physical activity breaks during classroom lessons; (3) 10 min/day physical activity homework. **Primary outcomes**: improve academic performance.	Socio-ecological conceptual framework.	10 min/day physical activity homework prepared by teachers.	=	There were no significant differences between groups for change in PA during school hours (*p* ≥ 0.303) or during the whole day (*p* ≥ 0.440). It did not affect their academic performance.
Salmon et al. [92] (2023)	Transform-Us! **Content**: The intervention consisted of (1) curriculum-based key learning messages, (2) interrupting classroom sitting time, (3) environmental cues and prompts, (4) newsletters, (5) homework assignments, (6) PA during recess and lunch breaks, and (7) teacher training. **Primary outcomes**: increase PA, reduce SB and decrease cardiometabolic risk factor scores.	SCT, behavioural choice theory and ecological systems theory.	Teachers prescribed PA homework.	+	At 18 months, there were intervention effects on children’s weekday SB (− 27 min, 95%CI: −47.3 to − 5.3) for the PA intervention, and on children’s average day PA (5.5 min, 95%CI: 0.1 to 10.8) for the SB intervention. At 30 months, there was an intervention effect for children’s average day SB (− 33.3 min, 95%CI: −50.6 and − 16.0) for the SB intervention.
Seljebotn et al. [93] (2019)	Active Schools. **Content**: Led by teachers and consisted of (1) physically active lessons, (2) physical active homework, and (3) physically active recess. **Primary outcomes**: increase PA and aerobic fitness.	NR	Physically active homework assigned by teachers (10 min/day).	+	Intervention effects were found for time in MVPA (adjusted mean difference of 8 min/day, 95% CI: 3.4–13, *p* < 0.001) and TPA (60 cpm, 95% CI: 15–105, *p* = 0.009).
Taylor et al. [94] (2018)	AS: Sk **Content**: It consisted of eight components: (1) active breaks, (2) bounce at the bell, (3) ‘Born To Move’ videos, (4) Daily Mile or 100 Mile Club, (5) playground activity challenge cards, (6) physical education teacher training, (7) newsletters, and (8) activity homework. **Primary outcomes**: increase PA and reduce SB.	Socioecological model.	Children received a homework pack which included a letter to parents and 10 different PA challenges. A separate pack of the individual challenges on small pieces of paper were also provided for children to take home if their original pack had been lost at home. Children received a weekly diary to complete whenever they had done PA at home. A blank class chart was provided to populate with names and update every week with school rewards for those who completed the most PA at home. Encouraged once daily.	=/+	Time spent engaged in SB during the school day was significantly less for the intervention children compared to the control group (− 9.0 min/day; *p* = 0.01). There were no intervention effects on any of the remaining outcome measures, although the trends for school day PA were in a favourable direction. The odds of achieving 30 min of MVPA per school day was 2.79 times higher in the intervention group compared to the control group, however this did not reach significance (*p* = 0.07).
Verloigne et al. [95] (2012)	UP4FUN **Content**: 6-week intervention consisting of (1) introduction of the project, (2) awareness of sitting time, (3) evaluation of sitting time, (4) influencing factors at home, (5) possibilities for activity breaks and active transportation, and (6) Family Fun Event. **Primary outcomes**: effect of intervention on SB.	Model of Planned Promotion for Population Health.	Weekly homework tasks given out via newsletter.	=	There were no significant differences in the change in sedentary time or LPA between intervention and control schools for the total sample or for the subgroup analyses by gender. However, children (specifically girls) in the intervention group had a higher decrease in MVPA than children in the control group. In the intervention group, children who lived with both parents and children with one or more siblings were less likely to reduce sedentary time after exposure to the intervention. Older children, girls and children who lived with both parents were less likely to increase LPA after the intervention

Abbreviations: PA, physical activity; SB, sedentary behaviour; TPA, total physical activity; MVPA, moderate-to-vigorous physical activity; LPA, light physical activity; MPA, moderate physical activity; VPA, vigorous physical activity; FMS, fundamental movement skills; BMI, body mass index; NR, not reported; SCT, social cognitive theory; SDT, self-determination theory; CPM, counts per minute; CI, confidence interval

^1^Intervention effectiveness relates to the main outcomes of the intervention, not the behavioural outcomes

^2^True Sleep: the amount of time between sleep start and sleep end

### Risk of bias Within Studies

The quality assessment revealed three studies had good quality [79, 86, 89], nine studies had fair quality [78, 80–82, 85, 88, 90, 92, 94] and seven had poor quality [83, 84, 87, 91, 93, 95, 96]. The main weaknesses were a lack of blinding of assessors and participants, high drop-out rates, and inadequate reporting surrounding the determination of sufficient sample size to ensure statistical significance with at least 80% power.

### Meta-analysis

**Fig. 2:**
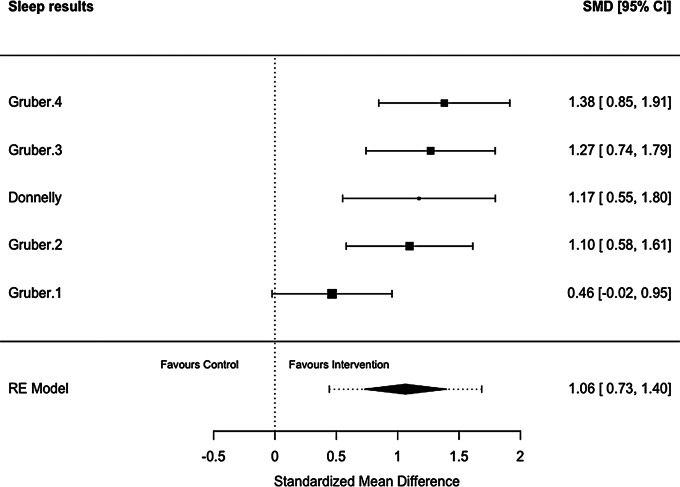
Forest plot of school-based interventions implementing homework on sleep expressed as standardised mean difference (SMD) with 95% confidence intervals (CIs). Legend: Each black square indicates an individual study’s SMD, with square size proportional to the study’s weight in the meta-analysis. Horizontal lines represent 95% CIs. The black diamond shape shows the overall effect estimate from the random-effects (RE) model, with the width indicating its 95% CI. The dotted vertical line indicates no effect (SMD = 0). Abbreviations: SMD, standardised mean difference; CI, confidence interval; RE model, Random Effects model.)

**Fig. 3:**
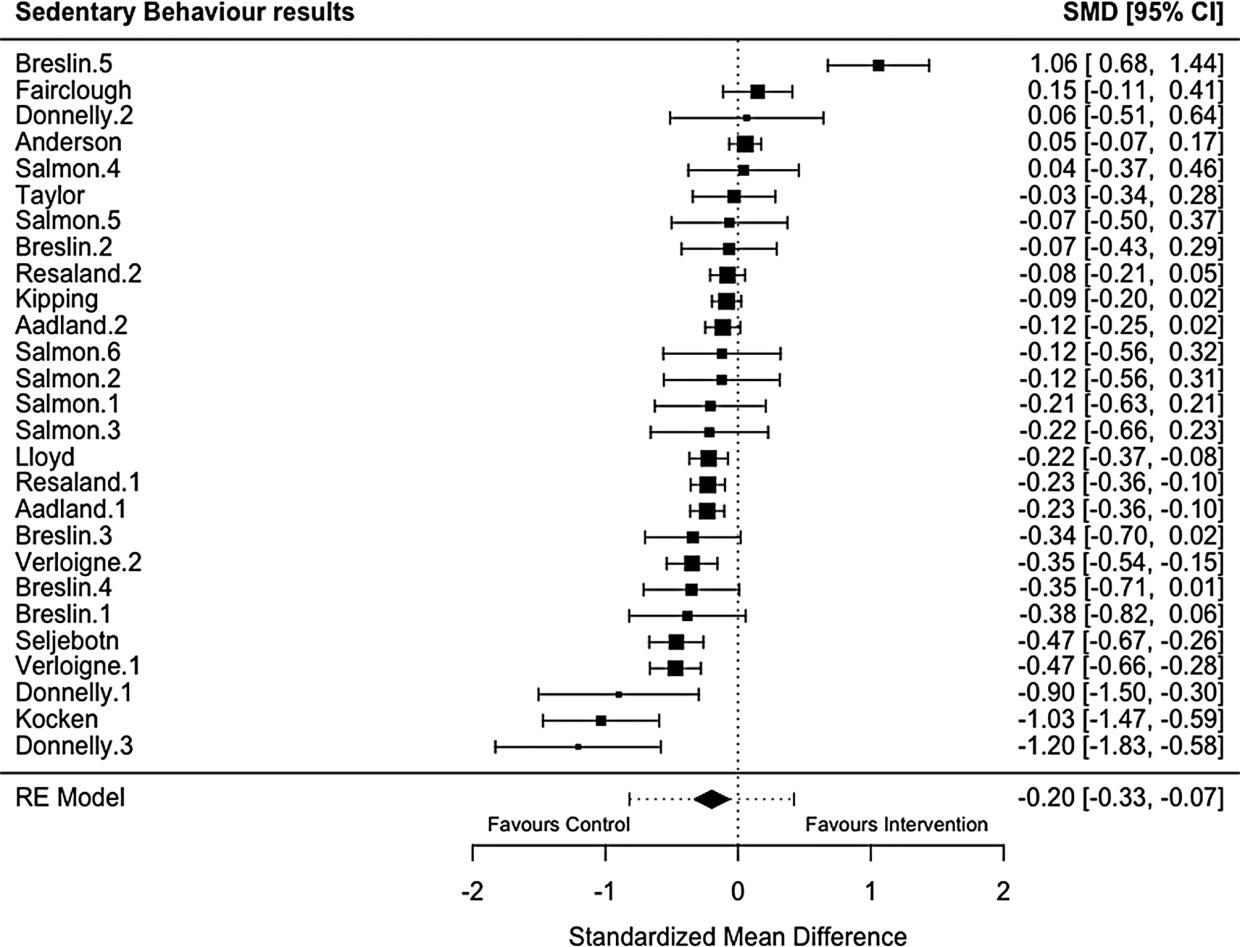
Forest plot of school-based interventions implementing homework on sedentary behaviour (SB) expressed as standardised mean difference (SMD) with 95% confidence intervals (CIs). Legend: Each black square indicates an individual study’s SMD, with square size proportional to the study’s weight in the meta-analysis. Horizontal lines represent 95% CIs. The black diamond shape shows the overall effect estimate from the random-effects (RE) model, with the width indicating its 95% CI. The dotted vertical line indicates no effect (SMD = 0). Abbreviations: SMD, standardised mean difference; CI, confidence interval; RE model, Random Effects model.)

**Fig. 4:**
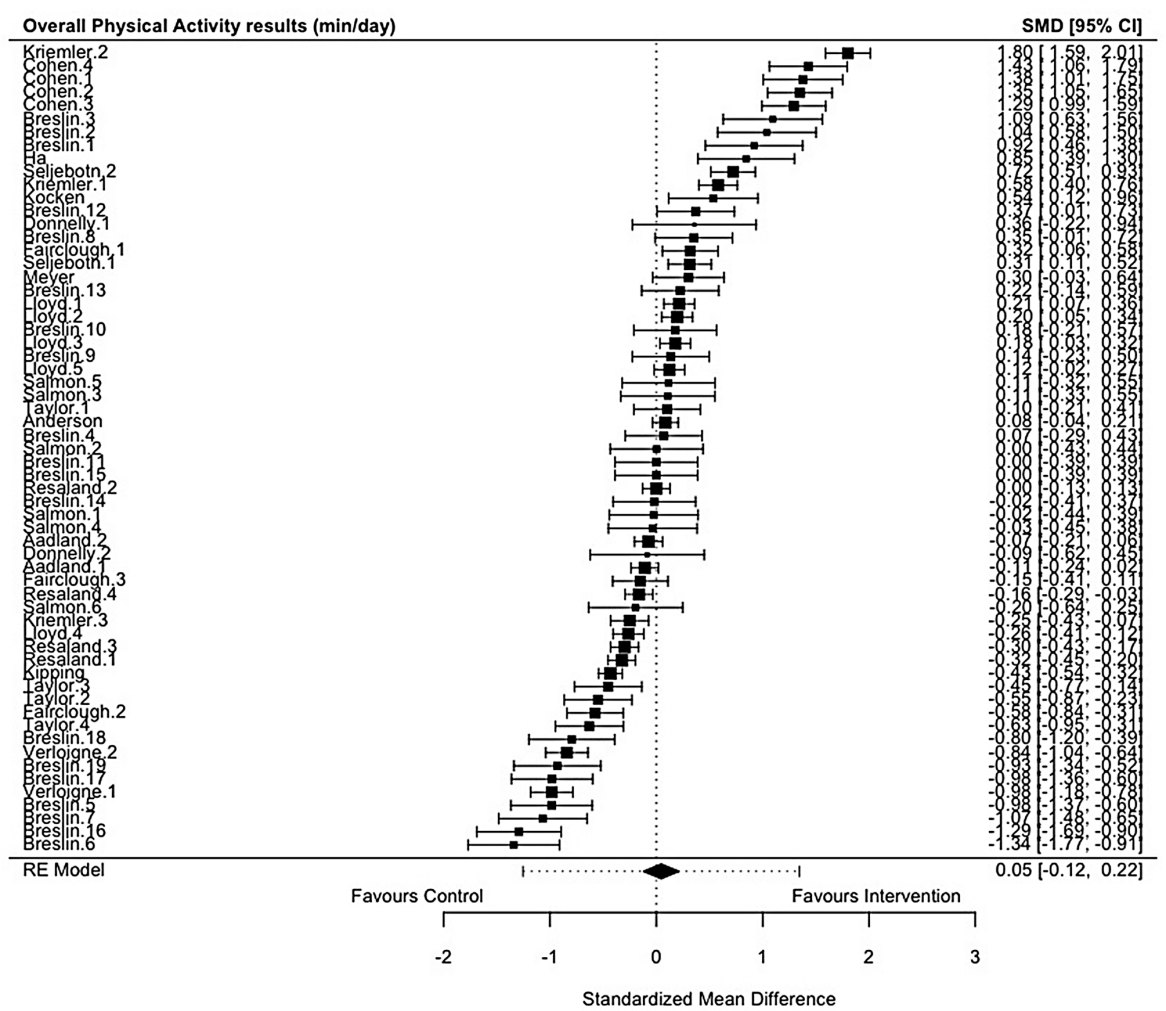
Forest plot of school-based interventions implementing homework on physical activity (PA)(min/day) expressed as standardised mean difference (SMD) with 95% confidence intervals (CIs). Legend: Each black square indicates an individual study’s SMD, with square size proportional to the study’s weight in the meta-analysis. Horizontal lines represent 95% CIs. The black diamond shape shows the overall effect estimate from the random-effects (RE) model, with the width indicating its 95% CI. The dotted vertical line indicates no effect (SMD = 0). Abbreviations: SMD, standardised mean difference; CI, confidence interval; RE model, Random Effects model.)

Of the 19 studies included in the review, 18 studies reported PA outcomes [78–83, 85–96], 13 studies reported SB outcomes [78–80, 82, 83, 86, 87, 89, 91–95], and two studies reported sleep outcomes [82, 84](Table 3). Meta-analyses demonstrated that school-based interventions which implemented homework produced significant improvements in sleep (Hedges’ *g* = 1.06, 95% CI = 0.73; 1.40, *p* < 0.0001; Fig. 2) and reductions in SB (Hedges’ *g* = -0.20, 95% CI = -0.33; -0.07, *p* = 0.0034; Fig. 3). Interventions examining PA, and its derivatives, showed no effects of the interventions compared to controls in the meta-analysis (Fig. 4; see supplementary file 5). Egger’s test for asymmetry was conducted for all behaviour outcomes with no significant indications of publication bias found (see supplementary file 6).

**Table 3:** Results of the eight meta-analyses examining movement behaviour outcomes from school-based interventions which implemented homework

Behaviour construct	No. cohorts	Length of INT (months)	Combined Participant (*N*): INT + CONT at follow-up	Mean age (years)^1^	Female (%)^2^	Summary effects				Heterogeneity		
						Hedges’ g	SE	95% CI	*P* value	Q	*P* value	I^2^ (%)
Sleep	5	1.5–2	334	8.82	56.06	1.0636	0.1707	0.7290; 1.3983	< 0.0001*	7.8215	0.0983	49.01
SB	27	1.5–30	9,736	9.06	53.26	-0.1984	0.0678	-0.3313; -0.0656	0.0034*	122.8859	< 0.0001	88.94
PA (cpm)	11	2–12	5,697	9.73	49.71	0.2156	0.1606	-0.0991; 0.5303	0.1794	219.6187	< 0.0001	96.95
PA (min/day)	61	1.5–30	19,737	9.39	52.02	0.0457	0.0870	-0.1247; 0.2162	0.5989	1297.9783	< 0.0001	97.05
MVPA (min/day)	29	1.5–30	11,454	9.65	52.98	0.2250	0.1304	-0.0305; 0.4805	0.0844	914.4162	< 0.0001	97.68
LPA (min/day)	11	2–10	3,921	9.72	50.04	0.0115	0.1686	-0.3189; 0.3418	0.9458	133.4622	< 0.0001	95.84
MPA (min/day)	7	3–9	1,531	9.47	49.76	-0.1422	0.2751	-0.6813; 0.3970	0.6053	95.1656	< 0.0001	95.56
VPA (min/day)	7	3–9	1,467	9.47	49.76	-0.1301	0.2847	-0.6681; 0.4280	0.6478	78.3245	< 0.0001	95.56

Abbreviations: INT, intervention; CONT, control, SB, sedentary behaviour; PA, physical activity; MVPA, moderate-to-vigorous physical activity; LPA, light physical activity; MPA, moderate physical activity; VPA, vigorous physical activity; CPM, counts per minute; SE, standard error; CI, confidence interval; p-value, probability value; Q, Cochran’s Q; I^2^, I-squared

^1^Calculated on baseline age and weighted by sample size reported by each study; ^2^ Based on entire sample at baseline, weighted by sample size; * p-value

Meta-regressions were conducted to assess the impact of intervention duration on behaviour constructs with intervention duration varying from 1.5 to 30 months (see Table 4). Longer intervention durations were seen to be more impactful for PA, measured in counts per minute (cpm), with a significant positive impact found (β = 0.14, *p* = 0.0241, CI = 0.02, 0.26). All other regressions were not significant (*p* > 0.005), meaning that intervention duration did not impact intervention effectiveness on behavioural outcomes. A meta-regression for sleep (β = 0.26, *p* = 0.68, CI = -1.70, 2.22) was conducted however, there was not enough range to establish a regression, and it was not reported within the table.

**Table 4:** Meta- regression of behaviour outcomes on length of intervention

Behaviour Outcome	Number of cohorts	Length of INT (months)	Overall backward elimination model			Single moderator model *P*
			Coefficient	*P*	95% CI	
SB	27	1.5–30	0.0045	0.0125	-0.0089; 0.0178	0.5137
PA (cpm)	11	2–12	0.1365	0.0834	0.0179; 0.2551	0.0241*
PA (min/day)	61	1.5–30	0.0170	0.4628	-0.0047; 0.0387	0.1247
MVPA	29	1.5–30	0.0050	0.4399	-0.0224; 0.0324	0.8206
LPA	11	2–10	0.0216	0.7961	-0.1080; 0.1512	0.7436
MPA	7	3–9	0.0350	0.6644	-0.2420; 0.3120	0.8044
VPA	7	3–9	0.0486	0.6259	-0.2368; 0.3339	0.7386

Abbreviations: INT, intervention; SB, sedentary behaviour; PA, physical activity; MVPA, moderate-to-vigorous physical activity; LPA, light physical activity; MPA, moderate physical activity; VPA, vigorous physical activity; CPM, counts per minute; CI, confidence interval; p, probability value

Note * Indicates statistical significance (*p* < 0.05)

### Subgroup Analysis

A summary of all subgroup analyses is provided in Table 5. The subgroups analysed within the context of each behaviour construct were the design of the study (RCT vs. non-RCT), theoretical framework (theory-based vs. non-theory-based), accelerometer placement (hip vs. wrist) and publication date (pre- vs. post-implementation of 24-hour movement guidelines).

Subgroup analysis showed for SB, RCT’s resulted in a significant effect but non-RCT’s did not (Table 5). However, between subgroup analysis was not statistically significant (*p* = 0.21). Within theory-based studies of SB, there was a significant effect, while a significant effect was also observed among non-theory-based studies (Table 5). Again, the difference between the two subgroups was not statistically significant (*p* = 0.70). Finally, subgroup analysis revealed a non-significant effect among studies conducted pre-implementation of 24-hour movement guidelines for SB. However, a significant effect was observed among studies conducted post-implementation of 24-hour movement guidelines (Table 5). The difference between the two time periods was not statistically significant (*p* = 0.53).

Q statistics for the remaining subgroup analyses for PA and sleep revealed no significant differences between subgroups and are detailed in Table 5.

(**Insert** Table 5: Sub-group analyses)

**Table 5:** Sub-group analyses

Behaviour construct	Subgroup Analysed		Effect Size (Hedges’ g)	Standard Mean Difference (95% CI)	*P* - value	Between Group Difference (*P* -value)
SB	Study design	RCT	-0.2228	(-0.3299, -0.1156)	< 0.0001	0.2124
		Non-RCT	-0.0158	(-0.5529, 0.5214)	0.9541	
	Theoretical framework	Theory-based	-0.1869	(-0.3527, -0.0265)	0.0227	0.7018
		Non-theory-based	-0.2426	(-0.3682, -0.1196)	0.0002	
	Accelerometer placement	Hip	*Insufficient variability across studies*			
		Wrist				
	Publication date	Pre-implementation of 24-hour movement guidelines	-0.1586	(-0.3937, 0.0766)	0.1863	0.5253
		Post-implementation of 24-hour movement guidelines	-0.2251	(-0.3266, -0.1236)	< 0.0001	
PA (cpm)	Study design	RCT	*All RCT*			
		Non-RCT				
	Theoretical framework	Theory-based	0.2343	(-0.1540, 0.6226)	0.2369	0.8208
		Non-theory-based	0.1364	(-0.1728, 0.4457)	0.3872	
	Accelerometer placement	Hip	*All hip*			
		Wrist				
	Publication date	Pre- implementation of 24-hour movement guidelines	0.2343	(-0.1540, 0.6226)	0.2369	0.8208
		Post-implementation of 24-hour movement guidelines	0.1364	(-0.1728, 0.4457)	0.3872	
PA (min/day)	Study design	RCT	0.1354	(-0.0533, 0.3240)	0.1596	0.1103
		Non-RCT	-0.1618	(-0.5111, 0.1874)	0.3637	
	Theoretical framework	Theory-based	0.0281	(-0.1708, 0.2271)	0.7815	0.6326
		Non-theory-based	0.1381	(-0.0432, 0.3194)	0.1354	
	Accelerometer placement	Hip	0.0629	(-0.1389, 0.2648)	0.5411	0.4547
		Wrist	-0.0968	(-0.3021, 0.1085)	0.3554	
	Publication date	Pre-implementation of 24-hour movement guidelines	0.0507	(-0.2046, 0.3059)	0.6972	0.9468
		Post-implementation of 24-hour movement guidelines	0.0370	(-0.1095, 0.1836)	0.6204	
MVPA	Study design	RCT	*All RCT*			
		Non-RCT				
	Theoretical framework	Theory-based	0.2518	(-0.0387, 0.5423)	0.0893	0.6247
		Non-theory-based	0.0637	(-0.3630, 0.4903)	0.7699	
	Accelerometer placement	Hip	0.2710	(-0.0221, 0.5640)	0.0699	0.2123
		Wrist	-0.2150	(-0.4863, 0.0564)	0.1205	
	Publication date	Pre-implementation of 24-hour movement guidelines	0.3978	(-0.0490, 0.8446)	0.0809	0.1689
		Post-implementation of 24-hour movement guidelines	0.0335	(-0.1602, 0.2273)	0.7345	
LPA	Study design	RCT	-0.0399	(-0.3177, 0.2379)	0.7784	0.9368
		Non-RCT	0.0816	(-0.6402, 0.8034)	0.8246	
	Theoretical framework	Theory-based	*Insufficient variability across studies*			
		Non-theory-based				
	Accelerometer placement	Hip	0.0419	(-0.3541, 0.4380)	0.8335	0.7259
		Wrist	-0.1123	(-0.7475, 0.5228)	0.7288	
	Publication date	Pre-implementation of 24-hour movement guidelines	0.0071	(-0.4385, 0.4528)	0.9749	0.9576
		Post-implementation of 24-hour movement guidelines	0.0358	(-0.4130, 0.4846)	0.8757	
MPA	Study design	RCT	-0.1907	(-0.9304, 0.5490)	0.6134	0.9085
		Non-RCT	-0.1183	(-0.8739, 0.6373)	0.7589	
	Theoretical framework	Theory-based	*Insufficient variability across studies*			
		Non-theory-based				
	Accelerometer placement	Hip	*Insufficient variability across studies*			
		Wrist				
	Publication date	Pre-implementation of 24-hour movement guidelines	*Insufficient variability across studies*			
		Post-implementation of 24-hour movement guidelines				
VPA	Study design	RCT	0.0085	(-0.2563, 0.2732)	0.9500	0.8068
		Non-RCT	-0.1790	(-0.9943, 0.6363)	0.6669	
	Theoretical framework	Theory-based	*Insufficient variability across studies*			
		Non-theory-based				
	Accelerometer placement	Hip	*Insufficient variability across studies*			
		Wrist				
	Publication date	Pre-implementation of 24-hour movement guidelines	*Insufficient variability across studies*			
		Post-implementation of 24-hour movement guidelines				

Abbreviations: SB, sedentary behaviour; PA, physical activity; MVPA, moderate-to-vigorous physical activity; LPA, light physical activity; MPA, moderate physical activity; VPA, vigorous physical activity; CPM, counts per minute; RCT, randomised controlled trial; non-RCT, non-randomised controlled trial; CI, confidence interval; p-value, probability value

## Discussion

This systematic review and meta-analysis aimed to: (1) assess the effectiveness of school-based health interventions which implement homework to improve 24-hour movement behaviours in primary school-aged children, and (2) examine moderating effects of study characteristics on intervention effectiveness (e.g., theory, study design, publication of 24-hour movement guidelines, time of day, accelerometer placement). To our knowledge, this is the first systematic review and meta-analysis examining such interventions using device-measured levels of PA, SB, and sleep. Nineteen studies met inclusion criteria for narrative synthesis and 104 movement behaviour cohorts were included in the quantitative analysis. Meta-analysis findings showed beneficial effects for SB (Hedges’ *g =* -0.20) and sleep (Hedges’ *g* = 1.06), but not for PA. These findings align with recent literature [98, 99] suggesting that school-based interventions may provide more favourable results if they focus more on reducing SB rather than promoting PA.

Schools are recognised as effective environments for PA promotion [98]. However, shifting the focus to reducing SB within schools may provide a more feasible and effective approach to improving movement behaviours in children. Some research, however, reports no improvements in PA or SB following intervention implementation [100]. Therefore, integrating homework into school-based interventions may reinforce health promoting behaviours at home, which may account for the greater reduction of SB observed in this study compared to previous reviews [101, 102]. As classroom-based interventions often yield inconsistent, short-terms effects, broader support systems involving teachers and parents may be crucial to achieve more consistent results, reinforcing health-promoting behaviours outside of school [103–107].

Within the included studies, homework was primarily supplementary to the intervention rather than the main focus. This made it challenging to isolate the specific direct effects of the homework related tasks upon movement behaviours. However, interventions including homework reported greater reductions in SB, suggesting that reinforcement of movement behaviours at home enhances effectiveness. Evidence suggests that without family involvement or reinforcement, long-term changes in children’s activity levels are unlikely to be sustained [86, 108]. Familial involvement, such as encouragement, logistical support, and co-participation, has been consistently associated with increased PA levels in children [109, 110]. Future interventions should prioritise homework as a primary method of intervention to better understanding its isolated effects on movement behaviours. This shift in focus may provide further insights into how homework-based interventions can be used to address other health related outcomes in children.

From Table 2, school-based interventions which implemented homework targeted additional primary outcomes including diet, FMS, BMI, and academic performance. However, few interventions specifically targeted sleep, despite those that did showing positive effects. A review of school-based interventions targeting 24-hour movement behaviours found only one study which considered sleep alongside PA and SB [111]. This study relied on a single baseline self-reported measure of sleep, pedometers for PA measurement, and self-reported SB [112]. Other school-based sleep interventions, were often ineffective and focused on adolescents rather than children [113, 114]. A review of short-term school-based sleep education interventions found minimal to no effects for improving sleep health, and highlighted the need for whole-school interventions to be trialled [115]. Methodological shortcomings including lack of theoretical frameworks and small sample sizes, may explain their limited success [116, 117], reinforcing the need for comprehensive, methodologically sound future approaches.

Our findings highlight the importance of a holistic approach to child health, integrating school and home environments. This aligns with the socio-ecological model, which hypothesises that behaviour change is most effective when multiple influences are addressed simultaneously [118]. Five included studies employed this model, with three reporting positive intervention outcomes [81, 88, 90]. Therefore, theory-informed homework involving both teachers and parents may extend the reach of school-based interventions and enhance behavioural change.

This idea is further supported by wider health promotion literature, which suggests that interventions grounded in theoretical models may produce a more sustained behaviour change [119–121]. In our analysis, eight interventions reporting positive outcomes were theory-based [80–83, 88, 92, 94, 96]. For example, the intervention by Taylor et al., [94] was informed by the socio-ecological model and reported significant reductions in SB but no significant intervention effects for PA. Despite encouragement for theory-informed interventions [122, 123], our subgroup analyses found no statistical differences between theory-based and non-theory-based interventions across all movement behaviour outcomes. This could be due to the low number of non-theory-based studies but may also be a consequence of poorly implemented theory in some interventions, where authors either failed to clearly outline the theoretical framework or simply referenced it without effectively integrating it into their intervention design. This aligns with existing literature, which suggests that the role of theory in youth PA interventions remains mixed [124], that authors fail to accurately report specifically how theory was used in intervention formulation [125, 126], contrasting with adult PA interventions where theory-based interventions are more effective [127, 128]. Future research should examine how theoretical constructs mediate the effects of multicomponent interventions on child movement behaviours.

The complexity of multicomponent interventions is further highlighted by the finding that intervention duration impacted PA outcomes but not SB or sleep. Longer interventions were more effective for PA (cpm), though caution is needed as this analysis did not account for collinear relationships between moderating behaviours (i.e., SB and sleep). Similar reviews report school-based interventions increasing PA during school hours but not out-of-school PA [129], suggesting that interventions may be insufficient for full-day activity promotion or may inadvertently contribute to a compensatory effect. The compensatory effect hypothesises that when PA in one domain or timespan increases, PA in another domain or timespan decreases which therefor maintains a constant level of PA [130]. Awareness of this effect is crucial in designing effective school-based PA interventions.

Extending school-based interventions beyond school hours through homework may enhance their impact. Studies using homework to improve movement behaviours report promising results. Two studies by Duncan et al., [131, 132] found significant intervention effects on step count during weekdays and weekends, though these studies were excluded due to pedometer use, rather than accelerometery, the gold standard for PA monitoring [133]. Despite positive effects, classroom-level randomisation, may introduce class contamination, affecting control participants [134]. Further research should use device-based measures (i.e., accelerometers), and address methodological limitations, such as class contamination, to better understand and enhance the use of homework’s role in movement behaviour interventions.

A further excluded study aimed at enhancing children’s sleep also used homework as a secondary intervention method [61]. Exclusion was due to reliance on subjective questionnaires. Chen et al., [61] concluded that school-based sleep education alone were insufficient in child behaviour change, and outlined that future studies should involve more active parental involvement which is supported by others [135–137]. This finding was inconsistent with previous child sleep interventions which outlined significant post-intervention improvements [138–140]. Such inconsistencies may be due to methodological differences such as small sample sizes [138–140], or targeting specific population groups (e.g., children with sleep disturbances) [138, 140]. Therefore, further studies which include sleep should look to involve parents within the intervention and aim to use device-based measures to help gain more reliable and consistent findings.

Our review highlights the complexity of promoting 24-hour movement behaviours in children and the importance of both the home and school environments. Teachers and parents receive various health promotion guidelines, yet rather than providing new recommendations, a paradigm shift may be needed. Instead of discouraging homework to reduce SB [141, 142], active homework could translate school-based interventions into the home. Active homework has been proposed as a direct method of increasing activity levels of school-children at the population level by increasing the time available to schools for shaping health behaviours [143, 144]. Additionally, parental modelling strongly influences child PA and SB [145]. Future interventions should consider how school-based interventions translate into home environments, reinforcing a holistic approach to behaviour change.

### Strengths and Limitations

This review has several strengths. Firstly, a comprehensive search strategy was undertaken across multiple databases to identify a range of potentially relevant studies. Another strength is the alignment with the PRISMA Statement which provides rigour and transparency in reporting on the conducted meta-analysis procedure (see supplementary file 1 for PRISMA checklist). All studies provided device-based measured estimates to provide gold standard measurements of each behaviour outcome. Only including device–based measures of PA, SB and sleep eliminated potential reporting and recall biases which provides more consistent data [146].

This meta-analysis has some limitations. Firstly, multiple outcomes from the same study were included in the meta-analyses. As such, more weight was given to these studies which can increase levels of heterogeneity considerably. This likely explains the moderate to high heterogeneity found in all meta-analyses, apart from sleep, (*I*^*2*^ = 88.85% to *I*^*2*^ = 97.68%). Secondly, we found a lack of transparency and inconsistencies in reporting methods which made it difficult to extract specific data. Study authors were contacted to provide further data or clarification, but two studies were not included because of poor reporting methods [66, 73]. Movement behaviours are multifaceted constructs and as such, more uniformity in reporting would strengthen these findings. Additionally, most interventions were short in duration (i.e., 3 months or less), with very few exploring the long-term intervention effects (i.e., >12 months). While attempts were made to explore sub-group differences, we were limited by the number of included studies. Future recommendations include encouraging authors to outline clear rigour criteria, such as blinding methods, to help assess risk of bias more accurately. A final key limitation of this review is the considerable heterogeneity in the homework interventions, including variations in content, delivery methods, and levels of parental involvement. As studies conducted in the home settings cannot verify who completes the assigned homework, especially when parental engagement might be low, this introduces uncertainty regarding intervention effectiveness. To address this, future studies should work towards creating a standardised definition and implementation framework for homework-based interventions. Additionally, developing robust measures to track engagement (i.e., such as teacher verification, digital monitoring tools, or reflective assessment), could improve the accuracy of evaluating intervention effectiveness.

## Conclusions

The results of the current review highlight a significant gap in primary-school-based interventions implementing homework which target all 24-hour movement behaviours. Our main findings highlight the beneficial effect of such interventions on SB and sleep outcomes. Beyond this, we highlight the lack of intervention programmes that include all three movement behaviours, particularly sleep. Our results suggest that researchers should focus more on reducing levels of SB and improving sleep; whilst examining the secondary effects on PA. This may lead to more substantial positive results rather than continuing with an emphasis placed on increasing PA.

## Electronic Supplementary Material

Below is the link to the electronic supplementary material.

Supplementary Material 1

Supplementary Material 2

Supplementary Material 3

Supplementary Material 4

Supplementary Material 5

Supplementary Material 6

Supplementary Material 7

## Data Availability

The datasets used and/or analysed during the current study are available from the corresponding author on reasonable request.

## Abbreviations

Abbreviation: Definition
BMI: Body mass index (kg/m2)
CI: Confidence intervals
CONT: Control
CPM: Counts per minute
FMS: Fundamental movement skills
Hours/day: Hours per day
INT: Intervention
LPA: Light physical activity
Mins: Minutes
Mins/day: Minutes per day
MPA: Moderate physical activity
MVPA: Moderate-to-vigorous physical activity
N: Sample size
NIH: National Health Institute
Non-RCT: Non-randomised controlled trial
NR: Not reported
PA: Physical activity
PICOS: Population, Intervention/Exposure, Comparison, Outcome and Study design
PRISMA: The Preferred Reporting Items for Systematic Reviews and Meta-Analyses
RCT: Randomised controlled trial
SB: Sedentary behaviour
SCT: Social cognitive theory
SD: Standard deviation
SDT: Self-determination theory
SMD: Standardised mean difference
TPA: Total physical activity
UK: United Kingdom
VPA: Vigorous physical activity
WHO: World Health Organisation
