# Supplementary material for: School-Based Homework Interventions for Improving 24-hour Movement Behaviours in Primary School Children: A Systematic Review and Meta-Analysis
Source: Sports Med Open. 2025 Aug 9;11:94. doi: 10.1186/s40798-025-00898-7 (PMC12335427; doi:10.1186/s40798-025-00898-7)
Supplement: Supplementary file 2 — Supplementary Material 2 [file 40798_2025_898_MOESM2_ESM.docx]

**School-based homework interventions for improving 24-hour movement behaviours in primary school children: A systematic review and meta-analysis.**

Sports Medicine – Open

April Forrest, ***Corresponding Author.***

University of the West of Scotland, School of Health and Life Sciences, Hamilton International Technology Park, Stephenson Place, Blantyre, Glasgow, G72 0LH, UK,

april.forrest@uws.ac.uk.

Dr Duncan Buchan.

University of the West of Scotland, School of Health and Life Sciences, Hamilton International Technology Park, Stephenson Place, Blantyre, Glasgow, G72 0LH, UK.

Professor Nicholas Sculthorpe.

University of the West of Scotland, School of Health and Life Sciences, Hamilton International Technology Park, Stephenson Place, Blantyre, Glasgow, G72 0LH, UK.

Dr Lawrence Hayes.

Lancaster Medical School, Faculty of Health & Medicine, Sir John Fisher Driver, Lancaster University, Lancaster, LA1 4AT, UK.

Dr Samantha Robinson.

University of the West of Scotland, School of Health and Life Sciences, Hamilton International Technology Park, Stephenson Place, Blantyre, Glasgow, G72 0LH, UK.

**Search Strategy**

- On 05/03/2024 an electronic literature search was conducted across six electronic databases which identified 2,275 potentially eligible articles: CINAHL Ultimate (EBSCO), PubMed, Scopus, SPORTDiscus (EBSCO), The Cochrane Library and Web of Science (core collection).
- Using the PICO (Population, Intervention, Comparison, and Outcome) framework, a comprehensive search strategy containing keywords and synonyms was developed with the assistance of a search specialist from the University library. This was done for each database searched.
- The screening of reference lists of retrieved articles was also performed, which yielded an additional 6 papers for inclusion.

**Table 1**

Summary of Keywords and Combinations Used for each Search.

| 1. | Child (population) |
| --- | --- |
| 2. | School setting |
| 3. | Homework |
| 4. | Physical Activity |
| 5. | Sedentary Behaviour |
| 6. | Sleep |
| 7. | 1 and 2 and 3 (intervention/exposure/phenomenon of interest) |
| 8. | 4 or 5 or 6 (outcomes) |
| 9. | 7 and 8 (results) |

**CINAHL Ultimate (896 results) – EBSCOHost Platform**

Notes:

- TI (title) or AB (abstract) or MH (subject heading) search fields were used.
- TX (all text) search field was used for ‘homework.’

| **#1** | TI ( Child* OR kid* OR "primary school age" OR schoolage OR school-age* OR school-age OR "elementary school age" OR basic school ) OR AB ( Child* OR kid* OR "primary school age" OR schoolage OR school-age* OR school-age OR "elementary school age" OR basic school ) OR MH ( Child* OR kid* OR "primary school age" OR schoolage OR school-age* OR school-age OR "elementary school age" OR basic school ) |
| --- | --- |
| **#2** | TI ( School setting OR "school based intervention" OR school-based intervention OR school based program* OR school program* OR school intervention OR school health intervention OR strateg* OR technique* OR intervention* OR program* OR health promotion* OR health education* OR health prevention OR health prevention program* OR health program* ) OR AB ( School setting OR "school based intervention" OR school-based intervention OR school based program* OR school program* OR school intervention OR school health intervention OR strateg* OR technique* OR intervention* OR program* OR health promotion* OR health education* OR health prevention OR health prevention program* OR health program* ) OR MH ( School setting OR "school based intervention" OR school-based intervention OR school based program* OR school program* OR school intervention OR school health intervention OR strateg* OR technique* OR intervention* OR program* OR health promotion* OR health education* OR health prevention OR health prevention program* OR health program* ) |
| **#3** | TX ( "homework" OR "home work" OR "home-work" ) |
| **#4** | TI ( ((Physical AND (activ* OR inactiv* OR total)) OR activ* OR walking OR aerobic exercise OR outdoor play OR exercise* OR motor behavio* OR movement) ) OR AB ( ((Physical AND (activ* OR inactiv* OR total)) OR activ* OR walking OR aerobic exercise OR outdoor play OR exercise* OR motor behavio* OR movement) ) OR MH ( ((Physical AND (activ* OR inactiv* OR total)) OR activ* OR walking OR aerobic exercise OR outdoor play OR exercise* OR motor behavio* OR movement) ) |
| **#5** | TI ( (sedentar* OR ((sitting OR computer OR media OR gaming OR TV OR television OR screen OR reading OR video) AND (behavio* OR time OR prolonged OR use))) ) OR AB ( (sedentar* OR ((sitting OR computer OR media OR gaming OR TV OR television OR screen OR reading OR video) AND (behavio* OR time OR prolonged OR use))) ) OR MH ( (sedentar* OR ((sitting OR computer OR media OR gaming OR TV OR television OR screen OR reading OR video) AND (behavio* OR time OR prolonged OR use))) ) |
| **#6** | TI ( (Sleep AND (behavio* OR duration OR quality OR pattern OR time OR disturbance OR health OR hygiene)) OR (sleep)) ) OR AB ( (Sleep AND (behavio* OR duration OR quality OR pattern OR time OR disturbance OR health OR hygiene)) OR (sleep)) ) OR MH ( (Sleep AND (behavio* OR duration OR quality OR pattern OR time OR disturbance OR health OR hygiene)) OR (sleep)) ) |
| **#7** | #1 AND #2 AND #3 |
| **#8** | #4 OR #5 OR #6 |
| **#9** | #7 AND #8 |

**PubMed (45 results)**

Notes:

- Title/Abstract (title, abstract, author keywords) search field used
- TW (text words) search field was used for ‘homework.’

| **#1** | "child*"[Title/Abstract] OR "kid"[Title/Abstract] OR "primary school age"[Title/Abstract] OR "schoolage"[Title/Abstract] OR "school age*"[Title/Abstract] OR "school-age"[Title/Abstract] OR "elementary school age"[Title/Abstract] OR "basic school"[Title/Abstract] |
| --- | --- |
| **#2** | "school setting"[Title/Abstract] OR "school based intervention"[Title/Abstract] OR "school based intervention"[Title/Abstract] OR "school based program*"[Title/Abstract] OR "school program*"[Title/Abstract] OR "school intervention"[Title/Abstract] OR "school health intervention"[Title/Abstract] OR "strateg*"[Title/Abstract] OR "technique*"[Title/Abstract] OR "intervention*"[Title/Abstract] OR "program*"[Title/Abstract] OR "health promotion*"[Title/Abstract] OR "health education*"[Title/Abstract] OR "health prevention"[Title/Abstract] OR "health prevention program*"[Title/Abstract] OR "health program*"[Title/Abstract] |
| **#3** | "homework"[Text Word] OR "home-work"[Text Word] OR "home-work"[Text Word] |
| **#4** | "Physical"[Title/Abstract] AND ("activ*"[Title/Abstract] OR "inactiv*"[Title/Abstract] OR "total"[Title/Abstract]) OR "activ*"[Title/Abstract] OR "walking"[Title/Abstract] OR "aerobic exercise"[Title/Abstract] OR "outdoor play"[Title/Abstract] OR "exercise*"[Title/Abstract] OR "motor behavio*"[Title/Abstract] OR "movement"[Title/Abstract] |
| **#5** | "sedentar*"[Title/Abstract] OR (("sitting"[Title/Abstract] OR "computer"[Title/Abstract] OR "media"[Title/Abstract] OR "gaming"[Title/Abstract] OR "TV"[Title/Abstract] OR "television"[Title/Abstract] OR "screen"[Title/Abstract] OR "reading"[Title/Abstract] OR "video"[Title/Abstract]) AND ("behavio*"[Title/Abstract] OR "time"[Title/Abstract] OR "prolonged"[Title/Abstract] OR "use"[Title/Abstract] |
| **#6** | Sleep[Title/Abstract] AND (behavio*[Title/Abstract] OR duration[Title/Abstract] OR quality[Title/Abstract] OR pattern[Title/Abstract] OR time[Title/Abstract] OR disturbance[Title/Abstract] OR health[Title/Abstract] OR hygiene[Title/Abstract])) OR (sleep[Title/Abstract]) |
| **#7** | #1 AND #2 AND #3 |
| **#8** | #4 OR #5 OR #6 |
| **#9** | #7 AND #8 |

**SCOPUS (11 results)**

Notes:

- TITLE-ABS-KEY (articles titles, abstract, keywords ) search field used.
- ALL (all fields) search field used for ‘homework.’

| **#1** | TITLE-ABS-KEY ( child* OR kid* OR "primary school age" OR schoolage OR school-age* OR school-age OR "elementary school age" OR basic AND school ) |
| --- | --- |
| **#2** | TITLE-ABS-KEY ( school AND setting OR "school based intervention" OR school-based AND intervention OR school AND based AND program* OR school AND program* OR school AND intervention OR school AND health AND intervention OR strateg* OR technique* OR intervention* OR program* OR health AND promotion* OR health AND education* OR health AND prevention OR health AND prevention AND program* OR health AND program*) |
| **#3** | ALL ( ( "homework" OR "home work" OR "home-work" ) ) |
| **#4** | TITLE-ABS-KEY ((( physical AND ( activ* OR inactiv* OR total ) ) OR activ* OR walking OR aerobic AND exercise OR outdoor AND play OR exercise* OR motor AND behavio* OR movement)) |
| **#5** | TITLE-ABS-KEY ( ( sedentar* OR ( ( sitting OR computer OR media OR gaming OR tv OR television OR screen OR reading OR video ) AND ( behavio* OR time OR prolonged OR use ) ) ) ) |
| **#6** | TITLE-ABS-KEY ( ( ( sleep AND ( behavio* OR duration OR quality OR pattern OR time OR disturbance OR health OR hygiene ) ) OR ( sleep ) ) ) |
| **#7** | #1 AND #2 AND #3 |
| **#8** | #4 OR #5 OR #6 |
| **#9** | #7 AND #8 |

**SPORTDiscus (356 results) – EBSCOHost Platform**

Notes:

- TI (title) or AB (abstract) or KW (keyword) search fields used.
- TX (all text) search field was used for ‘homework.’

| **#1** | AB (Child* OR kid* OR "primary school age" OR schoolage OR school-age* OR school-age OR "elementary school age" OR basic school) OR TI (Child* OR kid* OR "primary school age" OR schoolage OR school-age* OR school-age OR "elementary school age" OR basic school) OR KW (Child* OR kid* OR "primary school age" OR schoolage OR school-age* OR school-age OR "elementary school age" OR basic school) |
| --- | --- |
| **#2** | AB (School setting OR "school based intervention" OR school-based intervention OR school based program* OR school program* OR school intervention OR school health intervention OR strateg* OR technique* OR intervention* OR program* OR health promotion* OR health education* OR health prevention OR health prevention program* OR health program*) OR TI (School setting OR "school based intervention" OR school-based intervention OR school based program* OR school program* OR school intervention OR school health intervention OR strateg* OR technique* OR intervention* OR program* OR health promotion* OR health education* OR health prevention OR health prevention program* OR health program*) OR KW (School setting OR "school based intervention" OR school-based intervention OR school based program* OR school program* OR school intervention OR school health intervention OR strateg* OR technique* OR intervention* OR program* OR health promotion* OR health education* OR health prevention OR health prevention program* OR health program*) |
| **#3** | TX ("homework" OR "home work" OR "home-work") |
| **#4** | AB ((Physical AND (activ* OR inactiv* OR total)) OR activ* OR walking OR aerobic exercise OR outdoor play OR exercise* OR motor behavio* OR movement) OR TI((Physical AND (activ* OR inactiv* OR total)) OR activ* OR walking OR aerobic exercise OR outdoor play OR exercise* OR motor behavio* OR movement) OR KW((Physical AND (activ* OR inactiv* OR total)) OR activ* OR walking OR aerobic exercise OR outdoor play OR exercise* OR motor behavio* OR movement) |
| **#5** | AB (sedentar* OR ((sitting OR computer OR media OR gaming OR TV OR television OR screen OR reading OR video) AND (behavio* OR time OR prolonged OR use)) OR TI (sedentar* OR ((sitting OR computer OR media OR gaming OR TV OR television OR screen OR reading OR video) AND (behavio* OR time OR prolonged OR use)) OR KW (sedentar* OR ((sitting OR computer OR media OR gaming OR TV OR television OR screen OR reading OR video) AND (behavio* OR time OR prolonged OR use)) |
| **#6** | AB ((Sleep AND (behavio* OR duration OR quality OR pattern OR time OR disturbance OR health OR hygiene)) OR (sleep) OR TI ((Sleep AND (behavio* OR duration OR quality OR pattern OR time OR disturbance OR health OR hygiene)) OR (sleep) OR KW ((Sleep AND (behavio* OR duration OR quality OR pattern OR time OR disturbance OR health OR hygiene)) OR (sleep)) |
| **#7** | #1 AND #2 AND #3 |
| **#8** | #4 OR #5 OR #6 |
| **#9** | #7 AND #8 |

**Wiley Cochrane Library (388 results)**

Notes:

- Title Abstract Keywork (Record title, abstract and keywords) search field used.
- All Text (all available text) search field was used for ‘homework.’

| **#1** | (Child* OR kid* OR "primary school age" OR schoolage OR school-age* OR school-age OR "elementary school age" OR basic school):ti,ab,kw |
| --- | --- |
| **#2** | (School setting OR "school based intervention" OR school-based intervention OR school based program* OR school program* OR school intervention OR school health intervention OR strateg* OR technique* OR intervention* OR program* OR health promotion* OR health education* OR health prevention OR health prevention program* OR health program*):ti,ab,kw |
| **#3** | ("homework" OR "home work" OR "home-work") |
| **#4** | (((Physical AND (activ* OR inactiv* OR total)) OR activ* OR walking OR aerobic exercise OR outdoor play OR exercise* OR motor behavio* OR movement)):ti,ab,kw |
| **#5** | ((sedentar* OR ((sitting OR computer OR media OR gaming OR TV OR television OR screen OR reading OR video) AND (behavio* OR time OR prolonged OR use)))):ti,ab,kw |
| **#6** | ((Sleep AND (behavio* OR duration OR quality OR pattern OR time OR disturbance OR health OR hygiene)) OR (sleep)):ti,ab,kw |
| **#7** | #1 AND #2 AND #3 |
| **#8** | #4 OR #5 OR #6 |
| **#9** | #7 AND #8 |

**Web of Science (579 results) – Core Collection**

Notes:

- TS (topic) search field was used.

| **#1** | TS=(Child* OR kid* OR "primary school age" OR schoolage OR school-age* OR school-age OR "elementary school age" OR basic school) |
| --- | --- |
| **#2** | TS=(School setting OR "school based intervention" OR school-based intervention OR school based program* OR school program* OR school intervention OR school health intervention OR strateg* OR technique* OR intervention* OR program* OR health promotion* OR health education* OR health prevention OR health prevention program* OR health program*) |
| **#3** | TS=("homework" OR "home work" OR "home-work") |
| **#4** | TS=(((Physical AND (activ* OR inactiv* OR total)) OR activ* OR walking OR aerobic exercise OR outdoor play OR exercise* OR motor behavio* OR movement) ) |
| **#5** | TS=((sedentar* OR ((sitting OR computer OR media OR gaming OR TV OR television OR screen OR reading OR video) AND (behavio* OR time OR prolonged OR use)))) |
| **#6** | 6 TS=(((Sleep AND (behavio* OR duration OR quality OR pattern OR time OR disturbance OR health OR hygiene)) OR (sleep))) |
| **#7** | #1 AND #2 AND #3 |
| **#8** | #4 OR #5 OR #6 |
| **#9** | #7 AND #8 |
