# Supplementary material for: School-Based Homework Interventions for Improving 24-hour Movement Behaviours in Primary School Children: A Systematic Review and Meta-Analysis
Source: Sports Med Open. 2025 Aug 9;11:94. doi: 10.1186/s40798-025-00898-7 (PMC12335427; doi:10.1186/s40798-025-00898-7)
Supplement: Supplementary file 3 — Supplementary Material 3 [file 40798_2025_898_MOESM3_ESM.docx]

**School-based homework interventions for improving 24-hour movement behaviours in primary school children: A systematic review and meta-analysis.**

Sports Medicine – Open

April Forrest, ***Corresponding Author.***

University of the West of Scotland, School of Health and Life Sciences, Hamilton International Technology Park, Stephenson Place, Blantyre, Glasgow, G72 0LH, UK,

april.forrest@uws.ac.uk.

Dr Duncan Buchan.

University of the West of Scotland, School of Health and Life Sciences, Hamilton International Technology Park, Stephenson Place, Blantyre, Glasgow, G72 0LH, UK.

Professor Nicholas Sculthorpe.

University of the West of Scotland, School of Health and Life Sciences, Hamilton International Technology Park, Stephenson Place, Blantyre, Glasgow, G72 0LH, UK.

Dr Lawrence Hayes.

Lancaster Medical School, Faculty of Health & Medicine, Sir John Fisher Driver, Lancaster University, Lancaster, LA1 4AT, UK.

Dr Samantha Robinson.

University of the West of Scotland, School of Health and Life Sciences, Hamilton International Technology Park, Stephenson Place, Blantyre, Glasgow, G72 0LH, UK.

**Quality Assessment Tool for Controlled Intervention Studies.**

**Author (year):** Aadland *et al.,* (2019).

| **Criteria** | **Yes** | **No** | **Other** (CD, NR, NA) |
| --- | --- | --- | --- |
| 1. Was the study described as randomised, a randomised trial, a randomised clinical trial, or an RTC? | ✓ |  |  |
| 1. Was the method of randomisation adequate (i.e., use of randomly generate assignment)? | ✓ |  |  |
| 1. Was the treatment allocation concealed (so that assignments could not be predicted)? |  |  | NR |
| 1. Were study participants and providers blinded to treatment group assignment? |  |  | NR |
| 1. Were the people assessing the outcomes blinded to the participants’ group assignments? |  |  | NR |
| 1. Were the groups similar at baseline on important characteristics that could affect outcomes (e.g., demographics, risk factors, co-morbid conditions)? | ✓ |  |  |
| 1. Was the overall dropout rate from the study at its endpoint 20% or less than the number originally allocated to treatment? | ✓ |  |  |
| 1. Was the differential dropout rate between groups at the study’s endpoint 15% or less? | ✓ |  |  |
| 1. Was there high adherence to the intervention protocols for each treatment group? | ✓ |  |  |
| 1. Were other interventions avoided or similar in the groups (e.g., similar background treatments)? | ✓ |  |  |
| 1. Were outcomes assessed using valid and reliable measures, implemented consistently across all study participants? | ✓ |  |  |
| 1. Did the authors report the sample size was sufficiently large to be able to detect a difference in the main outcome between groups with at least 80% power? |  | ✓ |  |
| 1. Were outcomes reported or subgroups analysed prespecified (i.e., identified before analyses were conducted)? | ✓ |  |  |
| 1. Were all randomised participants analysed in the group to which they were originally assigned (i.e., did they used an intention-to-treat analysis)? | ✓ |  |  |

| **Quality Rating** (Good, Fair, Poor): 10 (fair) |
| --- |
| **Rater #1 Initials:** AF |
| **Rater #2 Initials:** SD |
| **Additional comments (if POOR, please state why):** |

Note: CD = cannot determine; NA= not applicable; NR = not reported.

**Quality Assessment Tool for Controlled Intervention Studies.**

**Author:** Anderson *et al.,* (2016)

| **Criteria** | **Yes** | **No** | **Other** (CD, NR, NA) |
| --- | --- | --- | --- |
| 1. Was the study described as randomised, a randomised trial, a randomised clinical trial, or an RTC? | ✓ |  |  |
| 1. Was the method of randomisation adequate (i.e., use of randomly generate assignment)? | ✓ |  |  |
| 1. Was the treatment allocation concealed (so that assignments could not be predicted)? | ✓ |  |  |
| 1. Were study participants and providers blinded to treatment group assignment? |  | ✓ |  |
| 1. Were the people assessing the outcomes blinded to the participants’ group assignments? | ✓ |  |  |
| 1. Were the groups similar at baseline on important characteristics that could affect outcomes (e.g., demographics, risk factors, co-morbid conditions)? | ✓ |  |  |
| 1. Was the overall dropout rate from the study at its endpoint 20% or less than the number originally allocated to treatment? |  | ✓ |  |
| 1. Was the differential dropout rate between groups at the study’s endpoint 15% or less? | ✓ |  |  |
| 1. Was there high adherence to the intervention protocols for each treatment group? | ✓ |  |  |
| 1. Were other interventions avoided or similar in the groups (e.g., similar background treatments)? | ✓ |  |  |
| 1. Were outcomes assessed using valid and reliable measures, implemented consistently across all study participants? | ✓ |  |  |
| 1. Did the authors report the sample size was sufficiently large to be able to detect a difference in the main outcome between groups with at least 80% power? | ✓ |  |  |
| 1. Were outcomes reported or subgroups analysed prespecified (i.e., identified before analyses were conducted)? | ✓ |  |  |
| 1. Were all randomised participants analysed in the group to which they were originally assigned (i.e., did they used an intention-to-treat analysis)? | ✓ |  |  |

| **Quality Rating** (Good, Fair, Poor): 12 (good) |
| --- |
| **Rater #1 Initials:** AF |
| **Rater #2 Initials:** SD |
| **Additional comments (if POOR, please state why):** |

Note: CD = cannot determine; NA= not applicable; NR = not reported.

**Quality Assessment Tool for Controlled Intervention Studies.**

**Author (year):** Breslin *et al.,* (2012)

| **Criteria** | **Yes** | **No** | **Other** (CD, NR, NA) |
| --- | --- | --- | --- |
| 1. Was the study described as randomised, a randomised trial, a randomised clinical trial, or an RTC? |  | ✓ |  |
| 1. Was the method of randomisation adequate (i.e., use of randomly generate assignment)? | ✓ |  |  |
| 1. Was the treatment allocation concealed (so that assignments could not be predicted)? | ✓ |  |  |
| 1. Were study participants and providers blinded to treatment group assignment? |  |  | NR |
| 1. Were the people assessing the outcomes blinded to the participants’ group assignments? |  |  | NR |
| 1. Were the groups similar at baseline on important characteristics that could affect outcomes (e.g., demographics, risk factors, co-morbid conditions)? | ✓ |  |  |
| 1. Was the overall dropout rate from the study at its endpoint 20% or less than the number originally allocated to treatment? | ✓ |  |  |
| 1. Was the differential dropout rate between groups at the study’s endpoint 15% or less? | ✓ |  |  |
| 1. Was there high adherence to the intervention protocols for each treatment group? | ✓ |  |  |
| 1. Were other interventions avoided or similar in the groups (e.g., similar background treatments)? | ✓ |  |  |
| 1. Were outcomes assessed using valid and reliable measures, implemented consistently across all study participants? | ✓ |  |  |
| 1. Did the authors report the sample size was sufficiently large to be able to detect a difference in the main outcome between groups with at least 80% power? |  | ✓ |  |
| 1. Were outcomes reported or subgroups analysed prespecified (i.e., identified before analyses were conducted)? | ✓ |  |  |
| 1. Were all randomised participants analysed in the group to which they were originally assigned (i.e., did they used an intention-to-treat analysis)? |  |  | NR |

| **Quality Rating** (Good, Fair, Poor): 9 (fair) |
| --- |
| **Rater #1 Initials:** AF |
| **Rater #2 Initials:** SD |
| **Additional comments (if POOR, please state why):** |

Note: CD = cannot determine; NA= not applicable; NR = not reported.

**Quality Assessment Tool for Controlled Intervention Studies.**

**Author (year):** Cohen *et al.,* (2015)

| **Criteria** | **Yes** | **No** | **Other** (CD, NR, NA) |
| --- | --- | --- | --- |
| 1. Was the study described as randomised, a randomised trial, a randomised clinical trial, or an RTC? | ✓ |  |  |
| 1. Was the method of randomisation adequate (i.e., use of randomly generate assignment)? | ✓ |  |  |
| 1. Was the treatment allocation concealed (so that assignments could not be predicted)? | ✓ |  |  |
| 1. Were study participants and providers blinded to treatment group assignment? |  | ✓ |  |
| 1. Were the people assessing the outcomes blinded to the participants’ group assignments? | ✓ |  |  |
| 1. Were the groups similar at baseline on important characteristics that could affect outcomes (e.g., demographics, risk factors, co-morbid conditions)? | ✓ |  |  |
| 1. Was the overall dropout rate from the study at its endpoint 20% or less than the number originally allocated to treatment? |  | ✓ |  |
| 1. Was the differential dropout rate between groups at the study’s endpoint 15% or less? |  | ✓ |  |
| 1. Was there high adherence to the intervention protocols for each treatment group? | ✓ |  |  |
| 1. Were other interventions avoided or similar in the groups (e.g., similar background treatments)? | ✓ |  |  |
| 1. Were outcomes assessed using valid and reliable measures, implemented consistently across all study participants? | ✓ |  |  |
| 1. Did the authors report the sample size was sufficiently large to be able to detect a difference in the main outcome between groups with at least 80% power? | ✓ |  |  |
| 1. Were outcomes reported or subgroups analysed prespecified (i.e., identified before analyses were conducted)? | ✓ |  |  |
| 1. Were all randomised participants analysed in the group to which they were originally assigned (i.e., did they used an intention-to-treat analysis)? | ✓ |  |  |

| **Quality Rating** (Good, Fair, Poor): 11 (fair) |
| --- |
| **Rater #1 Initials:** AF |
| **Rater #2 Initials:** SD |
| **Additional comments (if POOR, please state why):** |

Note: CD = cannot determine; NA= not applicable; NR = not reported.

**Quality Assessment Tool for Controlled Intervention Studies.**

**Author (year):** Donnelly *et al.,* (2023)

| **Criteria** | **Yes** | **No** | **Other** (CD, NR, NA) |
| --- | --- | --- | --- |
| 1. Was the study described as randomised, a randomised trial, a randomised clinical trial, or an RTC? | ✓ |  |  |
| 1. Was the method of randomisation adequate (i.e., use of randomly generate assignment)? |  |  | NR |
| 1. Was the treatment allocation concealed (so that assignments could not be predicted)? |  |  | NR |
| 1. Were study participants and providers blinded to treatment group assignment? | ✓ |  |  |
| 1. Were the people assessing the outcomes blinded to the participants’ group assignments? |  |  | NR |
| 1. Were the groups similar at baseline on important characteristics that could affect outcomes (e.g., demographics, risk factors, co-morbid conditions)? | ✓ |  |  |
| 1. Was the overall dropout rate from the study at its endpoint 20% or less than the number originally allocated to treatment? | ✓ |  |  |
| 1. Was the differential dropout rate between groups at the study’s endpoint 15% or less? | ✓ |  |  |
| 1. Was there high adherence to the intervention protocols for each treatment group? |  | ✓ |  |
| 1. Were other interventions avoided or similar in the groups (e.g., similar background treatments)? | ✓ |  |  |
| 1. Were outcomes assessed using valid and reliable measures, implemented consistently across all study participants? | ✓ |  |  |
| 1. Did the authors report the sample size was sufficiently large to be able to detect a difference in the main outcome between groups with at least 80% power? |  | ✓ |  |
| 1. Were outcomes reported or subgroups analysed prespecified (i.e., identified before analyses were conducted)? | ✓ |  |  |
| 1. Were all randomised participants analysed in the group to which they were originally assigned (i.e., did they used an intention-to-treat analysis)? | ✓ |  |  |

| **Quality Rating** (Good, Fair, Poor): 9 (fair) |
| --- |
| **Rater #1 Initials:** AF |
| **Rater #2 Initials:** SD |
| **Additional comments (if POOR, please state why):** |

Note: CD = cannot determine; NA= not applicable; NR = not reported.

**Quality Assessment Tool for Controlled Intervention Studies.**

**Author (year):** Fairclough *et al.,*(2013)

| **Criteria** | **Yes** | **No** | **Other** (CD, NR, NA) |
| --- | --- | --- | --- |
| 1. Was the study described as randomised, a randomised trial, a randomised clinical trial, or an RTC? | ✓ |  |  |
| 1. Was the method of randomisation adequate (i.e., use of randomly generate assignment)? | ✓ |  |  |
| 1. Was the treatment allocation concealed (so that assignments could not be predicted)? |  | ✓ |  |
| 1. Were study participants and providers blinded to treatment group assignment? |  | ✓ |  |
| 1. Were the people assessing the outcomes blinded to the participants’ group assignments? |  | ✓ |  |
| 1. Were the groups similar at baseline on important characteristics that could affect outcomes (e.g., demographics, risk factors, co-morbid conditions)? | ✓ |  |  |
| 1. Was the overall dropout rate from the study at its endpoint 20% or less than the number originally allocated to treatment? | ✓ |  |  |
| 1. Was the differential dropout rate between groups at the study’s endpoint 15% or less? | ✓ |  |  |
| 1. Was there high adherence to the intervention protocols for each treatment group? |  |  | NR |
| 1. Were other interventions avoided or similar in the groups (e.g., similar background treatments)? | ✓ |  |  |
| 1. Were outcomes assessed using valid and reliable measures, implemented consistently across all study participants? | ✓ |  |  |
| 1. Did the authors report the sample size was sufficiently large to be able to detect a difference in the main outcome between groups with at least 80% power? |  | ✓ |  |
| 1. Were outcomes reported or subgroups analysed prespecified (i.e., identified before analyses were conducted)? | ✓ |  |  |
| 1. Were all randomised participants analysed in the group to which they were originally assigned (i.e., did they used an intention-to-treat analysis)? |  |  | NR |

| **Quality Rating** (Good, Fair, Poor): 8 (poor) |
| --- |
| **Rater #1 Initials:** AF |
| **Rater #2 Initials:** SD |
| **Additional comments (if POOR, please state why):**  Poor blinding practices, processes surrounding adherence and analyses not reported. |

Note: CD = cannot determine; NA= not applicable; NR = not reported.

**Quality Assessment Tool for Controlled Intervention Studies.**

**Author (year):** Goran and Reynolds (2005)

| **Criteria** | **Yes** | **No** | **Other** (CD, NR, NA) |
| --- | --- | --- | --- |
| 1. Was the study described as randomised, a randomised trial, a randomised clinical trial, or an RTC? | ✓ |  |  |
| 1. Was the method of randomisation adequate (i.e., use of randomly generate assignment)? |  |  | NR |
| 1. Was the treatment allocation concealed (so that assignments could not be predicted)? |  |  | NR |
| 1. Were study participants and providers blinded to treatment group assignment? |  |  | NR |
| 1. Were the people assessing the outcomes blinded to the participants’ group assignments? |  |  | NR |
| 1. Were the groups similar at baseline on important characteristics that could affect outcomes (e.g., demographics, risk factors, co-morbid conditions)? | ✓ |  |  |
| 1. Was the overall dropout rate from the study at its endpoint 20% or less than the number originally allocated to treatment? | ✓ |  |  |
| 1. Was the differential dropout rate between groups at the study’s endpoint 15% or less? | ✓ |  |  |
| 1. Was there high adherence to the intervention protocols for each treatment group? |  |  | NR |
| 1. Were other interventions avoided or similar in the groups (e.g., similar background treatments)? | ✓ |  |  |
| 1. Were outcomes assessed using valid and reliable measures, implemented consistently across all study participants? | ✓ |  |  |
| 1. Did the authors report the sample size was sufficiently large to be able to detect a difference in the main outcome between groups with at least 80% power? |  |  | NR |
| 1. Were outcomes reported or subgroups analysed prespecified (i.e., identified before analyses were conducted)? | ✓ |  |  |
| 1. Were all randomised participants analysed in the group to which they were originally assigned (i.e., did they used an intention-to-treat analysis)? | ✓ |  |  |

| **Quality Rating** (Good, Fair, Poor): 8 (poor) |
| --- |
| **Rater #1 Initials:** AF |
| **Rater #2 Initials:** SD |
| **Additional comments (if POOR, please state why):** |

Note: CD = cannot determine; NA= not applicable; NR = not reported.

**Quality Assessment Tool for Controlled Intervention Studies.**

**Author (year):** Gruber *et al.,*(2016)

| **Criteria** | **Yes** | **No** | **Other** (CD, NR, NA) |
| --- | --- | --- | --- |
| 1. Was the study described as randomised, a randomised trial, a randomised clinical trial, or an RTC? |  | ✓ |  |
| 1. Was the method of randomisation adequate (i.e., use of randomly generate assignment)? |  | ✓ |  |
| 1. Was the treatment allocation concealed (so that assignments could not be predicted)? |  | ✓ |  |
| 1. Were study participants and providers blinded to treatment group assignment? |  | ✓ |  |
| 1. Were the people assessing the outcomes blinded to the participants’ group assignments? |  | ✓ |  |
| 1. Were the groups similar at baseline on important characteristics that could affect outcomes (e.g., demographics, risk factors, co-morbid conditions)? | ✓ |  |  |
| 1. Was the overall dropout rate from the study at its endpoint 20% or less than the number originally allocated to treatment? | ✓ |  |  |
| 1. Was the differential dropout rate between groups at the study’s endpoint 15% or less? | ✓ |  |  |
| 1. Was there high adherence to the intervention protocols for each treatment group? |  |  | NR |
| 1. Were other interventions avoided or similar in the groups (e.g., similar background treatments)? | ✓ |  |  |
| 1. Were outcomes assessed using valid and reliable measures, implemented consistently across all study participants? | ✓ |  |  |
| 1. Did the authors report the sample size was sufficiently large to be able to detect a difference in the main outcome between groups with at least 80% power? |  |  | NR |
| 1. Were outcomes reported or subgroups analysed prespecified (i.e., identified before analyses were conducted)? | ✓ |  |  |
| 1. Were all randomised participants analysed in the group to which they were originally assigned (i.e., did they used an intention-to-treat analysis)? |  |  | NR |

| **Quality Rating** (Good, Fair, Poor): 6 (poor) |
| --- |
| **Rater #1 Initials:** AF |
| **Rater #2 Initials:** SD |
| **Additional comments (if POOR, please state why):**  Was not a randomised trial (non-randomised trail), and did not report on adherence, sample size power and intention to treat analyses. |

Note: CD = cannot determine; NA= not applicable; NR = not reported.

**Quality Assessment Tool for Controlled Intervention Studies.**

**Author (year):** Ha *et al.,*(2021)

| **Criteria** | **Yes** | **No** | **Other** (CD, NR, NA) |
| --- | --- | --- | --- |
| 1. Was the study described as randomised, a randomised trial, a randomised clinical trial, or an RTC? | ✓ |  |  |
| 1. Was the method of randomisation adequate (i.e., use of randomly generate assignment)? | ✓ |  |  |
| 1. Was the treatment allocation concealed (so that assignments could not be predicted)? | ✓ |  |  |
| 1. Were study participants and providers blinded to treatment group assignment? |  |  | NR |
| 1. Were the people assessing the outcomes blinded to the participants’ group assignments? |  | ✓ |  |
| 1. Were the groups similar at baseline on important characteristics that could affect outcomes (e.g., demographics, risk factors, co-morbid conditions)? | ✓ |  |  |
| 1. Was the overall dropout rate from the study at its endpoint 20% or less than the number originally allocated to treatment? |  | ✓ |  |
| 1. Was the differential dropout rate between groups at the study’s endpoint 15% or less? | ✓ |  |  |
| 1. Was there high adherence to the intervention protocols for each treatment group? | ✓ |  |  |
| 1. Were other interventions avoided or similar in the groups (e.g., similar background treatments)? | ✓ |  |  |
| 1. Were outcomes assessed using valid and reliable measures, implemented consistently across all study participants? | ✓ |  |  |
| 1. Did the authors report the sample size was sufficiently large to be able to detect a difference in the main outcome between groups with at least 80% power? | ✓ |  |  |
| 1. Were outcomes reported or subgroups analysed prespecified (i.e., identified before analyses were conducted)? | ✓ |  |  |
| 1. Were all randomised participants analysed in the group to which they were originally assigned (i.e., did they used an intention-to-treat analysis)? |  |  | NR |

| **Quality Rating** (Good, Fair, Poor): 10 (fair) |
| --- |
| **Rater #1 Initials:** AF |
| **Rater #2 Initials:** SD |
| **Additional comments (if POOR, please state why):** |

Note: CD = cannot determine; NA= not applicable; NR = not reported.

**Quality Assessment Tool for Controlled Intervention Studies.**

**Author (year):** Kipping *et al.,*(2014)

| **Criteria** | **Yes** | **No** | **Other** (CD, NR, NA) |
| --- | --- | --- | --- |
| 1. Was the study described as randomised, a randomised trial, a randomised clinical trial, or an RTC? | ✓ |  |  |
| 1. Was the method of randomisation adequate (i.e., use of randomly generate assignment)? | ✓ |  |  |
| 1. Was the treatment allocation concealed (so that assignments could not be predicted)? | ✓ |  |  |
| 1. Were study participants and providers blinded to treatment group assignment? |  | ✓ |  |
| 1. Were the people assessing the outcomes blinded to the participants’ group assignments? | ✓ |  |  |
| 1. Were the groups similar at baseline on important characteristics that could affect outcomes (e.g., demographics, risk factors, co-morbid conditions)? | ✓ |  |  |
| 1. Was the overall dropout rate from the study at its endpoint 20% or less than the number originally allocated to treatment? | ✓ |  |  |
| 1. Was the differential dropout rate between groups at the study’s endpoint 15% or less? | ✓ |  |  |
| 1. Was there high adherence to the intervention protocols for each treatment group? |  |  | NR |
| 1. Were other interventions avoided or similar in the groups (e.g., similar background treatments)? | ✓ |  |  |
| 1. Were outcomes assessed using valid and reliable measures, implemented consistently across all study participants? | ✓ |  |  |
| 1. Did the authors report the sample size was sufficiently large to be able to detect a difference in the main outcome between groups with at least 80% power? | ✓ |  |  |
| 1. Were outcomes reported or subgroups analysed prespecified (i.e., identified before analyses were conducted)? | ✓ |  |  |
| 1. Were all randomised participants analysed in the group to which they were originally assigned (i.e., did they used an intention-to-treat analysis)? | ✓ |  |  |

| **Quality Rating** (Good, Fair, Poor): 12 (good) |
| --- |
| **Rater #1 Initials:** AF |
| **Rater #2 Initials:** SD |
| **Additional comments (if POOR, please state why):** |

Note: CD = cannot determine; NA= not applicable; NR = not reported.

**Quality Assessment Tool for Controlled Intervention Studies.**

**Author (year):** Kocken *et al.,*(2016)

| **Criteria** | **Yes** | **No** | **Other** (CD, NR, NA) |
| --- | --- | --- | --- |
| 1. Was the study described as randomised, a randomised trial, a randomised clinical trial, or an RTC? | ✓ |  |  |
| 1. Was the method of randomisation adequate (i.e., use of randomly generate assignment)? |  |  | NR |
| 1. Was the treatment allocation concealed (so that assignments could not be predicted)? |  |  | NR |
| 1. Were study participants and providers blinded to treatment group assignment? |  |  | NR |
| 1. Were the people assessing the outcomes blinded to the participants’ group assignments? |  |  | NR |
| 1. Were the groups similar at baseline on important characteristics that could affect outcomes (e.g., demographics, risk factors, co-morbid conditions)? | ✓ |  |  |
| 1. Was the overall dropout rate from the study at its endpoint 20% or less than the number originally allocated to treatment? | ✓ |  |  |
| 1. Was the differential dropout rate between groups at the study’s endpoint 15% or less? | ✓ |  |  |
| 1. Was there high adherence to the intervention protocols for each treatment group? | ✓ |  |  |
| 1. Were other interventions avoided or similar in the groups (e.g., similar background treatments)? | ✓ |  |  |
| 1. Were outcomes assessed using valid and reliable measures, implemented consistently across all study participants? | ✓ |  |  |
| 1. Did the authors report the sample size was sufficiently large to be able to detect a difference in the main outcome between groups with at least 80% power? |  | ✓ |  |
| 1. Were outcomes reported or subgroups analysed prespecified (i.e., identified before analyses were conducted)? | ✓ |  |  |
| 1. Were all randomised participants analysed in the group to which they were originally assigned (i.e., did they used an intention-to-treat analysis)? |  |  | NR |

| **Quality Rating** (Good, Fair, Poor): 8 (poor) |
| --- |
| **Rater #1 Initials:** AF |
| **Rater #2 Initials:** SD |
| **Additional comments (if POOR, please state why):**  Poor reporting on randomisation processes, determining sufficient power size and analyses. |

Note: CD = cannot determine; NA= not applicable; NR = not reported.

**Quality Assessment Tool for Controlled Intervention Studies.**

**Author (year):** Kriemler *et al.,*(2010)

| **Criteria** | **Yes** | **No** | **Other** (CD, NR, NA) |
| --- | --- | --- | --- |
| 1. Was the study described as randomised, a randomised trial, a randomised clinical trial, or an RTC? | ✓ |  |  |
| 1. Was the method of randomisation adequate (i.e., use of randomly generate assignment)? | ✓ |  |  |
| 1. Was the treatment allocation concealed (so that assignments could not be predicted)? | ✓ |  |  |
| 1. Were study participants and providers blinded to treatment group assignment? |  |  | NR |
| 1. Were the people assessing the outcomes blinded to the participants’ group assignments? | ✓ |  |  |
| 1. Were the groups similar at baseline on important characteristics that could affect outcomes (e.g., demographics, risk factors, co-morbid conditions)? | ✓ |  |  |
| 1. Was the overall dropout rate from the study at its endpoint 20% or less than the number originally allocated to treatment? |  |  | NR |
| 1. Was the differential dropout rate between groups at the study’s endpoint 15% or less? |  |  | NR |
| 1. Was there high adherence to the intervention protocols for each treatment group? |  | ✓ |  |
| 1. Were other interventions avoided or similar in the groups (e.g., similar background treatments)? | ✓ |  |  |
| 1. Were outcomes assessed using valid and reliable measures, implemented consistently across all study participants? | ✓ |  |  |
| 1. Did the authors report the sample size was sufficiently large to be able to detect a difference in the main outcome between groups with at least 80% power? | ✓ |  |  |
| 1. Were outcomes reported or subgroups analysed prespecified (i.e., identified before analyses were conducted)? | ✓ |  |  |
| 1. Were all randomised participants analysed in the group to which they were originally assigned (i.e., did they used an intention-to-treat analysis)? | ✓ |  |  |

| **Quality Rating** (Good, Fair, Poor): 10 (fair) |
| --- |
| **Rater #1 Initials:** AF |
| **Rater #2 Initials:** SD |
| **Additional comments (if POOR, please state why):** |

Note: CD = cannot determine; NA= not applicable; NR = not reported.

**Quality Assessment Tool for Controlled Intervention Studies.**

**Author (year):** Lloyd *et al.,*(2018)

| **Criteria** | **Yes** | **No** | **Other** (CD, NR, NA) |
| --- | --- | --- | --- |
| 1. Was the study described as randomised, a randomised trial, a randomised clinical trial, or an RTC? | ✓ |  |  |
| 1. Was the method of randomisation adequate (i.e., use of randomly generate assignment)? | ✓ |  |  |
| 1. Was the treatment allocation concealed (so that assignments could not be predicted)? | ✓ |  |  |
| 1. Were study participants and providers blinded to treatment group assignment? |  | ✓ |  |
| 1. Were the people assessing the outcomes blinded to the participants’ group assignments? | ✓ |  |  |
| 1. Were the groups similar at baseline on important characteristics that could affect outcomes (e.g., demographics, risk factors, co-morbid conditions)? | ✓ |  |  |
| 1. Was the overall dropout rate from the study at its endpoint 20% or less than the number originally allocated to treatment? | ✓ |  |  |
| 1. Was the differential dropout rate between groups at the study’s endpoint 15% or less? | ✓ |  |  |
| 1. Was there high adherence to the intervention protocols for each treatment group? |  |  | NR |
| 1. Were other interventions avoided or similar in the groups (e.g., similar background treatments)? | ✓ |  |  |
| 1. Were outcomes assessed using valid and reliable measures, implemented consistently across all study participants? | ✓ |  |  |
| 1. Did the authors report the sample size was sufficiently large to be able to detect a difference in the main outcome between groups with at least 80% power? | ✓ |  |  |
| 1. Were outcomes reported or subgroups analysed prespecified (i.e., identified before analyses were conducted)? | ✓ |  |  |
| 1. Were all randomised participants analysed in the group to which they were originally assigned (i.e., did they used an intention-to-treat analysis)? | ✓ |  |  |

| **Quality Rating** (Good, Fair, Poor): 12 (good) |
| --- |
| **Rater #1 Initials:** AF |
| **Rater #2 Initials:** SD |
| **Additional comments (if POOR, please state why):** |

Note: CD = cannot determine; NA= not applicable; NR = not reported.

**Quality Assessment Tool for Controlled Intervention Studies.**

**Author (year):** Meyer *et al.,*(2014)

| **Criteria** | **Yes** | **No** | **Other** (CD, NR, NA) |
| --- | --- | --- | --- |
| 1. Was the study described as randomised, a randomised trial, a randomised clinical trial, or an RTC? | ✓ |  |  |
| 1. Was the method of randomisation adequate (i.e., use of randomly generate assignment)? | ✓ |  |  |
| 1. Was the treatment allocation concealed (so that assignments could not be predicted)? | ✓ |  |  |
| 1. Were study participants and providers blinded to treatment group assignment? |  |  | CD |
| 1. Were the people assessing the outcomes blinded to the participants’ group assignments? | ✓ |  |  |
| 1. Were the groups similar at baseline on important characteristics that could affect outcomes (e.g., demographics, risk factors, co-morbid conditions)? | ✓ |  |  |
| 1. Was the overall dropout rate from the study at its endpoint 20% or less than the number originally allocated to treatment? |  | ✓ |  |
| 1. Was the differential dropout rate between groups at the study’s endpoint 15% or less? | ✓ |  |  |
| 1. Was there high adherence to the intervention protocols for each treatment group? |  |  | NR |
| 1. Were other interventions avoided or similar in the groups (e.g., similar background treatments)? | ✓ |  |  |
| 1. Were outcomes assessed using valid and reliable measures, implemented consistently across all study participants? | ✓ |  |  |
| 1. Did the authors report the sample size was sufficiently large to be able to detect a difference in the main outcome between groups with at least 80% power? | ✓ |  |  |
| 1. Were outcomes reported or subgroups analysed prespecified (i.e., identified before analyses were conducted)? | ✓ |  |  |
| 1. Were all randomised participants analysed in the group to which they were originally assigned (i.e., did they used an intention-to-treat analysis)? |  | ✓ |  |

| **Quality Rating** (Good, Fair, Poor): 10 (fair) |
| --- |
| **Rater #1 Initials:** AF |
| **Rater #2 Initials:** SD |
| **Additional comments (if POOR, please state why):** |

Note: CD = cannot determine; NA= not applicable; NR = not reported.

**Quality Assessment Tool for Controlled Intervention Studies.**

**Author (year):** Resaland *et al.,*(2016)

| **Criteria** | **Yes** | **No** | **Other** (CD, NR, NA) |
| --- | --- | --- | --- |
| 1. Was the study described as randomised, a randomised trial, a randomised clinical trial, or an RTC? | ✓ |  |  |
| 1. Was the method of randomisation adequate (i.e., use of randomly generate assignment)? |  |  | NR |
| 1. Was the treatment allocation concealed (so that assignments could not be predicted)? |  |  | NR |
| 1. Were study participants and providers blinded to treatment group assignment? |  |  | NR |
| 1. Were the people assessing the outcomes blinded to the participants’ group assignments? |  |  | NR |
| 1. Were the groups similar at baseline on important characteristics that could affect outcomes (e.g., demographics, risk factors, co-morbid conditions)? | ✓ |  |  |
| 1. Was the overall dropout rate from the study at its endpoint 20% or less than the number originally allocated to treatment? |  |  | NR |
| 1. Was the differential dropout rate between groups at the study’s endpoint 15% or less? |  |  | NR |
| 1. Was there high adherence to the intervention protocols for each treatment group? | ✓ |  |  |
| 1. Were other interventions avoided or similar in the groups (e.g., similar background treatments)? | ✓ |  |  |
| 1. Were outcomes assessed using valid and reliable measures, implemented consistently across all study participants? | ✓ |  |  |
| 1. Did the authors report the sample size was sufficiently large to be able to detect a difference in the main outcome between groups with at least 80% power? |  |  | NR |
| 1. Were outcomes reported or subgroups analysed prespecified (i.e., identified before analyses were conducted)? | ✓ |  |  |
| 1. Were all randomised participants analysed in the group to which they were originally assigned (i.e., did they used an intention-to-treat analysis)? | ✓ |  |  |

| **Quality Rating** (Good, Fair, Poor): 7 (poor) |
| --- |
| **Rater #1 Initials:** AF |
| **Rater #2 Initials:** SD |
| **Additional comments (if POOR, please state why):**  Poor reporting on randomisation processes, determining sufficient power size and drop-out rates (only outlined drop out during follow up but not in relation to which group and numbers at completion). |

Note: CD = cannot determine; NA= not applicable; NR = not reported.

**Quality Assessment Tool for Controlled Intervention Studies.**

**Author (year):** Salmon *et al.,*(2023)

| **Criteria** | **Yes** | **No** | **Other** (CD, NR, NA) |
| --- | --- | --- | --- |
| 1. Was the study described as randomised, a randomised trial, a randomised clinical trial, or an RTC? | ✓ |  |  |
| 1. Was the method of randomisation adequate (i.e., use of randomly generate assignment)? | ✓ |  |  |
| 1. Was the treatment allocation concealed (so that assignments could not be predicted)? | ✓ |  |  |
| 1. Were study participants and providers blinded to treatment group assignment? |  | ✓ |  |
| 1. Were the people assessing the outcomes blinded to the participants’ group assignments? |  | ✓ |  |
| 1. Were the groups similar at baseline on important characteristics that could affect outcomes (e.g., demographics, risk factors, co-morbid conditions)? | ✓ |  |  |
| 1. Was the overall dropout rate from the study at its endpoint 20% or less than the number originally allocated to treatment? |  |  | NR |
| 1. Was the differential dropout rate between groups at the study’s endpoint 15% or less? |  |  | NR |
| 1. Was there high adherence to the intervention protocols for each treatment group? |  |  | NR |
| 1. Were other interventions avoided or similar in the groups (e.g., similar background treatments)? | ✓ |  |  |
| 1. Were outcomes assessed using valid and reliable measures, implemented consistently across all study participants? | ✓ |  |  |
| 1. Did the authors report the sample size was sufficiently large to be able to detect a difference in the main outcome between groups with at least 80% power? | ✓ |  |  |
| 1. Were outcomes reported or subgroups analysed prespecified (i.e., identified before analyses were conducted)? | ✓ |  |  |
| 1. Were all randomised participants analysed in the group to which they were originally assigned (i.e., did they used an intention-to-treat analysis)? | ✓ |  |  |

| **Quality Rating** (Good, Fair, Poor): 9 (fair) |
| --- |
| **Rater #1 Initials:** AF |
| **Rater #2 Initials:** SD |
| **Additional comments (if POOR, please state why):** |

Note: CD = cannot determine; NA= not applicable; NR = not reported.

**Quality Assessment Tool for Controlled Intervention Studies.**

**Author (year):** Seljebotn *et al.,*(2019)

| **Criteria** | **Yes** | **No** | **Other** (CD, NR, NA) |
| --- | --- | --- | --- |
| 1. Was the study described as randomised, a randomised trial, a randomised clinical trial, or an RTC? | ✓ |  |  |
| 1. Was the method of randomisation adequate (i.e., use of randomly generate assignment)? | ✓ |  |  |
| 1. Was the treatment allocation concealed (so that assignments could not be predicted)? | ✓ |  |  |
| 1. Were study participants and providers blinded to treatment group assignment? |  |  | NR |
| 1. Were the people assessing the outcomes blinded to the participants’ group assignments? |  |  | NR |
| 1. Were the groups similar at baseline on important characteristics that could affect outcomes (e.g., demographics, risk factors, co-morbid conditions)? | ✓ |  |  |
| 1. Was the overall dropout rate from the study at its endpoint 20% or less than the number originally allocated to treatment? |  |  | NR |
| 1. Was the differential dropout rate between groups at the study’s endpoint 15% or less? |  |  | NR |
| 1. Was there high adherence to the intervention protocols for each treatment group? |  |  | NR |
| 1. Were other interventions avoided or similar in the groups (e.g., similar background treatments)? | ✓ |  |  |
| 1. Were outcomes assessed using valid and reliable measures, implemented consistently across all study participants? | ✓ |  |  |
| 1. Did the authors report the sample size was sufficiently large to be able to detect a difference in the main outcome between groups with at least 80% power? | ✓ |  |  |
| 1. Were outcomes reported or subgroups analysed prespecified (i.e., identified before analyses were conducted)? | ✓ |  |  |
| 1. Were all randomised participants analysed in the group to which they were originally assigned (i.e., did they used an intention-to-treat analysis)? |  | ✓ |  |

| **Quality Rating** (Good, Fair, Poor): 8 (poor) |
| --- |
| **Rater #1 Initials:** AF |
| **Rater #2 Initials:** SD |
| **Additional comments (if POOR, please state why):**  Poor reporting on group blinding and assessor blinding, as well as on participant drop-rates, and analyses. |

Note: CD = cannot determine; NA= not applicable; NR = not reported.

**Quality Assessment Tool for Controlled Intervention Studies.**

**Author (year):** Taylor *et al.,*(2018)

| **Criteria** | **Yes** | **No** | **Other** (CD, NR, NA) |
| --- | --- | --- | --- |
| 1. Was the study described as randomised, a randomised trial, a randomised clinical trial, or an RTC? | ✓ |  |  |
| 1. Was the method of randomisation adequate (i.e., use of randomly generate assignment)? | ✓ |  |  |
| 1. Was the treatment allocation concealed (so that assignments could not be predicted)? |  | ✓ |  |
| 1. Were study participants and providers blinded to treatment group assignment? |  | ✓ |  |
| 1. Were the people assessing the outcomes blinded to the participants’ group assignments? |  | ✓ |  |
| 1. Were the groups similar at baseline on important characteristics that could affect outcomes (e.g., demographics, risk factors, co-morbid conditions)? | ✓ |  |  |
| 1. Was the overall dropout rate from the study at its endpoint 20% or less than the number originally allocated to treatment? |  | ✓ |  |
| 1. Was the differential dropout rate between groups at the study’s endpoint 15% or less? | ✓ |  |  |
| 1. Was there high adherence to the intervention protocols for each treatment group? | ✓ |  |  |
| 1. Were other interventions avoided or similar in the groups (e.g., similar background treatments)? | ✓ |  |  |
| 1. Were outcomes assessed using valid and reliable measures, implemented consistently across all study participants? | ✓ |  |  |
| 1. Did the authors report the sample size was sufficiently large to be able to detect a difference in the main outcome between groups with at least 80% power? | ✓ |  |  |
| 1. Were outcomes reported or subgroups analysed prespecified (i.e., identified before analyses were conducted)? | ✓ |  |  |
| 1. Were all randomised participants analysed in the group to which they were originally assigned (i.e., did they used an intention-to-treat analysis)? |  |  | NR |

| **Quality Rating** (Good, Fair, Poor): 9 (fair) |
| --- |
| **Rater #1 Initials:**  AF |
| **Rater #2 Initials:** SD |
| **Additional comments (if POOR, please state why):** |

Note: CD = cannot determine; NA= not applicable; NR = not reported.

**Quality Assessment Tool for Controlled Intervention Studies.**

**Author (year):** Verloigne *et al.,*(2012)

| **Criteria** | **Yes** | **No** | **Other** (CD, NR, NA) |
| --- | --- | --- | --- |
| 1. Was the study described as randomised, a randomised trial, a randomised clinical trial, or an RTC? | ✓ |  |  |
| 1. Was the method of randomisation adequate (i.e., use of randomly generate assignment)? | ✓ |  |  |
| 1. Was the treatment allocation concealed (so that assignments could not be predicted)? | ✓ |  |  |
| 1. Were study participants and providers blinded to treatment group assignment? |  | ✓ |  |
| 1. Were the people assessing the outcomes blinded to the participants’ group assignments? |  | ✓ |  |
| 1. Were the groups similar at baseline on important characteristics that could affect outcomes (e.g., demographics, risk factors, co-morbid conditions)? | ✓ |  |  |
| 1. Was the overall dropout rate from the study at its endpoint 20% or less than the number originally allocated to treatment? |  | ✓ |  |
| 1. Was the differential dropout rate between groups at the study’s endpoint 15% or less? |  | ✓ |  |
| 1. Was there high adherence to the intervention protocols for each treatment group? |  |  | NR |
| 1. Were other interventions avoided or similar in the groups (e.g., similar background treatments)? | ✓ |  |  |
| 1. Were outcomes assessed using valid and reliable measures, implemented consistently across all study participants? | ✓ |  |  |
| 1. Did the authors report the sample size was sufficiently large to be able to detect a difference in the main outcome between groups with at least 80% power? |  |  | NR |
| 1. Were outcomes reported or subgroups analysed prespecified (i.e., identified before analyses were conducted)? | ✓ |  |  |
| 1. Were all randomised participants analysed in the group to which they were originally assigned (i.e., did they used an intention-to-treat analysis)? |  | ✓ |  |

| **Quality Rating** (Good, Fair, Poor): 6 (poor) |
| --- |
| **Rater #1 Initials:** AF |
| **Rater #2 Initials:** SD |
| **Additional comments (if POOR, please state why):**  Poor reporting regarding sample size power and adherence rates. Blinding practices were not followed and there was a high drop-out rate. |

Note: CD = cannot determine; NA= not applicable; NR = not reported.
