# Supplementary material for: School-Based Homework Interventions for Improving 24-hour Movement Behaviours in Primary School Children: A Systematic Review and Meta-Analysis
Source: Sports Med Open. 2025 Aug 9;11:94. doi: 10.1186/s40798-025-00898-7 (PMC12335427; doi:10.1186/s40798-025-00898-7)
Supplement: Supplementary file 4 — Supplementary Material 4 [file 40798_2025_898_MOESM4_ESM.docx]

**School-based homework interventions for improving 24-hour movement behaviours in primary school children: A systematic review and meta-analysis.**

Sports Medicine – Open

April Forrest, ***Corresponding Author.***

University of the West of Scotland, School of Health and Life Sciences, Hamilton International Technology Park, Stephenson Place, Blantyre, Glasgow, G72 0LH, UK,

april.forrest@uws.ac.uk.

Dr Duncan Buchan.

University of the West of Scotland, School of Health and Life Sciences, Hamilton International Technology Park, Stephenson Place, Blantyre, Glasgow, G72 0LH, UK.

Professor Nicholas Sculthorpe.

University of the West of Scotland, School of Health and Life Sciences, Hamilton International Technology Park, Stephenson Place, Blantyre, Glasgow, G72 0LH, UK.

Dr Lawrence Hayes.

Lancaster Medical School, Faculty of Health & Medicine, Sir John Fisher Driver, Lancaster University, Lancaster, LA1 4AT, UK.

Dr Samantha Robinson.

University of the West of Scotland, School of Health and Life Sciences, Hamilton International Technology Park, Stephenson Place, Blantyre, Glasgow, G72 0LH, UK.

**Supplementary File 4. Excluded Studies.**

| **Publication** | **Reason for exclusion** |
| --- | --- |
| Aarts et al., 2012 (1) | Wrong population |
| Adams et al., 2017 (2) | Wrong population |
| Al‐Khudairy et al., 2017 (3) | Wrong population |
| Ali et al., 2022 (4) | Wrong population |
| Arundell et al., 2016 (5) | Wrong study design |
| Arundell et al., 2020 (6) | Not accelerometer measured |
| Atkin et al., 2011 (7) | Wrong study design |
| Barnes et al., 2015 (8) | Not school-based |
| Birnkammer & Calvano, 2023 (9) | Wrong population |
| Centeio et al., 2014 (10) | No intentional homework |
| Chen et al., 2023 (11) | Not accelerometer measured |
| Cohen et al., 2015 (12) | Duplicate data |
| Duncan et al., 2011 (13) | Not accelerometer measured |
| Eather, Morgan, & Lubans, 2013 (14) | Not accelerometer measured |
| Esteban-Cornejo et al., 2015 (15) | Wrong population |
| Hartmann et al., 2010 (16) | Data unavailable |
| Kipping, Jago, & Lawlor, 2012 (17) | Wrong study design |
| Kobel et al., 2017 (18) | Not accelerometer measured |
| Kvalø et al., 2017 (19) | Not accelerometer measured |
| Lonsdale et al., 2016 (20) | Wrong study design |
| Lubans et al., 2014 (21) | Duplicate data |
| Malden et al., 2018 (22) | Wrong population |
| Morris et al., 2013 (23) | Data unavailable |
| Roth et al., 2015 (24) | Wrong population |
| Siegrist et al., 2013 (25) | Not accelerometer measured |
| Veldman et al., 2020 (26) | No intentional homework |
| Verloigne et al., 2015 (27) | Duplicate data |

**References**

1. Aarts PB, Hartingsveldt M, Anderson PG, Tillaar I, Burg J, Geurts A, et al. The Pirate Group Intervention Protocol: Description and a Case Report of a Modified Constraint-induced Movement Therapy Combined with Bimanual Training for Young Children with Unilateral Spastic Cerebral Palsy. Occup Ther Int. 2012;19(2):76-87.

2. Adams ILJ, Smits-Engelsman B, Lust JM, Wilson PH, Steenbergen B. Feasibility of Motor Imagery Training for Children with Developmental Coordination Disorder - A Pilot Study. Front Psychol. 2017;8.

3. Al‐Khudairy L, Loveman E, Colquitt JL, Mead E, Johnson RE, Fraser H, et al. Diet, physical activity and behavioural interventions for the treatment of overweight or obese adolescents aged 12 to 17 years. Cochrane Database Syst Rev. 2017(6).

4. Ali N, Mukhtar S, Khan Y, Ahmad M, Khan ZU. Analysis of secondary school students’ academic performance and parental involvement in children education at home. Educ Sci J. 2022;24(9):118-42.

5. Arundell L, Fletcher E, Salmon J, Veitch J, Hinkley T. A systematic review of the prevalence of sedentary behavior during the after-school period among children aged 5-18 years. Int J Behav Nutr Phys Act. 2016;13:1-9.

6. Arundell L, Parker K, Timperio A, Salmon J, Veitch J. Home-based screen time behaviors amongst youth and their parents: familial typologies and their modifiable correlates. BMC Public Health. 2020;20(1):1492-.

7. Atkin A, Gorely T, Biddle S, Cavill N, Foster C. Interventions to Promote Physical Activity in Young People Conducted in the Hours Immediately After School: A Systematic Review. Int J Behav Med. 2011;18(3):176-87.

8. Barnes AT, Plotnikoff RC, Collins CE, Morgan PJ. Feasibility and Preliminary Efficacy of the MADE4Life Program: A Pilot Randomized Controlled Trial. J Phys Act Health. 2015;12(10):1378-93.

9. Birnkammer S, Calvano C. A Creative and Movement-Based Blended Intervention for Children in Outpatient Residential Care: A Mixed-Method, Multi-Center, Single-Arm Feasibility Trial. Children (Basel). 2023;10(2):207.

10. Centeio EE, Somers C, McCaughtry N, Shen B, Gutuskey L, Martin JJ, et al. Physical Activity Change Through Comprehensive School Physical Activity Programs in Urban Elementary Schools. J Teach Phys Educ. 2014;33(4):573-91.

11. Chen S-J, Li SX, Zhang J-H, Lam SP, Yu M, Man yW, et al. School-Based Sleep Education Program for Children: A Cluster Randomized Controlled Trial. Healthcare (Basel). 2023;11(13):1853.

12. Cohen KE, Morgan PJ, Plotnikoff RC, Barnett LM, Lubans DR. Improvements in fundamental movement skill competency mediate the effect of the SCORES intervention on physical activity and cardiorespiratory fitness in children. J Sport Sci. 2015;33(18):1908-18.

13. Duncan S, McPhee JC, Schluter PJ, Zinn C, Smith R, Schofield G. Efficacy of a compulsory homework programme for increasing physical activity and healthy eating in children: the healthy homework pilot study. Int J Behav Nutr Phys Act. 2011;8:127.

14. Eather N, Morgan P, Lubans D. Feasibility and preliminary efficacy of the Fit4Fun intervention for improving physical fitness in a sample of primary school children: a pilot study. Phys Educ Sport Pedagogy. 2013;18(4):389-411.

15. Esteban-Cornejo I, Martinez-Gomez D, Sallis JF, Cabanas-Sánchez V, Fernández-Santos J, Castro-Piñero J, et al. Objectively measured and self-reported leisure-time sedentary behavior and academic performance in youth: The UP&DOWN Study. Prev Med. 2015;77:106-11.

16. Hartmann T, Zahner L, Pühse U, Schneider S, Puder JJ, Kriemler S. Physical activity, bodyweight, health and fear of negative evaluation in primary school children. Scand J Med Sci Sports. 2010;20(1):1-e34.

17. Kipping RR, Jago R, Lawlor DA. Developing parent involvement in a school-based child obesity prevention intervention: a qualitative study and process evaluation. J Public Health. 2012;34(2):236-44.

18. Kobel S, Lämmle C, Wartha O, Kesztyüs D, Wirt T, Steinacker J. Effects of a Randomised Controlled School-Based Health Promotion Intervention on Obesity Related Behavioural Outcomes of Children with Migration Background. J Immigr Minor Health. 2017;19(2):254-62.

19. Kvalø SE, Bru E, Brønnick K, Dyrstad SM. Does increased physical activity in school affect children's executive function and aerobic fitness? Scand J Med Sci Sports. 2017;27(12):1833-41.

20. Lonsdale C, ers T, Cohen KE, Parker P, Noetel M, Hartwig T, et al. Scaling-up an efficacious school-based physical activity intervention: Study protocol for the 'Internet-based Professional Learning to help teachers support Activity in Youth' (iPLAY) cluster randomized controlled trial and scale-up implementation evaluation. BMC Public Health. 2016;16.

21. Lubans D, Cohen K, Plotnikoff R, Callister R, Morgan P. The SCORES physical activity intervention for children attending schools in low-income communities: a cluster RCT. J Sci Med Sport. 2014;18:e121.

22. Malden S, Reilly J, Gibson AM, Hughes A. Adaptation of the ToyBox pre-school obesity prevention programme for use in Scotland: intervention adaptation processes and baseline results of a feasibility cluster randomised controlled trial. Obes Facts. 2018;11:213.

23. Morris J, Gorely T, Sedgwick M, Nevill A, Nevill M. Effect of the Great Activity Programme on healthy lifestyle behaviours in 7–11 year olds. J Sports Sci. 2013;31(12):1280-93.

24. Roth K, Kriemler S, Lehmacher W, Ruf KC, Graf C, Hebestreit H. Effects of a Physical Activity Intervention in Preschool Children. Med Sci Sports Exerc. 2015;47(12):2542-51.

25. Siegrist M, Lammel C, Haller B, Christle J, Halle M. Effects of a physical education program on physical activity, fitness, and health in children: The Juven TUM project. Scand J Med Sci Sports. 2013;23(3):323-30.

26. Veldman SLC, Jones RA, Stanley RM, Cliff DP, Vella SA, Howard SJ, et al. Promoting Physical Activity and Executive Functions Among Children: A Cluster Randomized Controlled Trial of an After-School Program in Australia. J Phys Act Health. 2020;17(10):940-6.

27. Verloigne M, Ridgers ND, Chinapaw M, Altenburg T, Bere E, Berntsen S, et al. The UP4FUN Intervention Effect on Breaking Up Sedentary Time in 10- to 12-Year-Old Belgian Children: The ENERGY Project. Pediatr Exerc Sci. 2015;27(2):234-42.
