# Supplementary material for: School-Based Homework Interventions for Improving 24-hour Movement Behaviours in Primary School Children: A Systematic Review and Meta-Analysis
Source: Sports Med Open. 2025 Aug 9;11:94. doi: 10.1186/s40798-025-00898-7 (PMC12335427; doi:10.1186/s40798-025-00898-7)
Supplement: Supplementary file 5 — Supplementary Material 5 [file 40798_2025_898_MOESM5_ESM.docx]

**School-based homework interventions for improving 24-hour movement behaviours in primary school children: A systematic review and meta-analysis.**

Sports Medicine – Open

April Forrest, ***Corresponding Author.***

University of the West of Scotland, School of Health and Life Sciences, Hamilton International Technology Park, Stephenson Place, Blantyre, Glasgow, G72 0LH, UK,

april.forrest@uws.ac.uk.

Dr Duncan Buchan.

University of the West of Scotland, School of Health and Life Sciences, Hamilton International Technology Park, Stephenson Place, Blantyre, Glasgow, G72 0LH, UK.

Professor Nicholas Sculthorpe.

University of the West of Scotland, School of Health and Life Sciences, Hamilton International Technology Park, Stephenson Place, Blantyre, Glasgow, G72 0LH, UK.

Dr Lawrence Hayes.

Lancaster Medical School, Faculty of Health & Medicine, Sir John Fisher Driver, Lancaster University, Lancaster, LA1 4AT, UK.

Dr Samantha Robinson.

University of the West of Scotland, School of Health and Life Sciences, Hamilton International Technology Park, Stephenson Place, Blantyre, Glasgow, G72 0LH, UK.

**Supplementary File 5: Forest Plots**

**Physical Activity (cpm) forest plot**
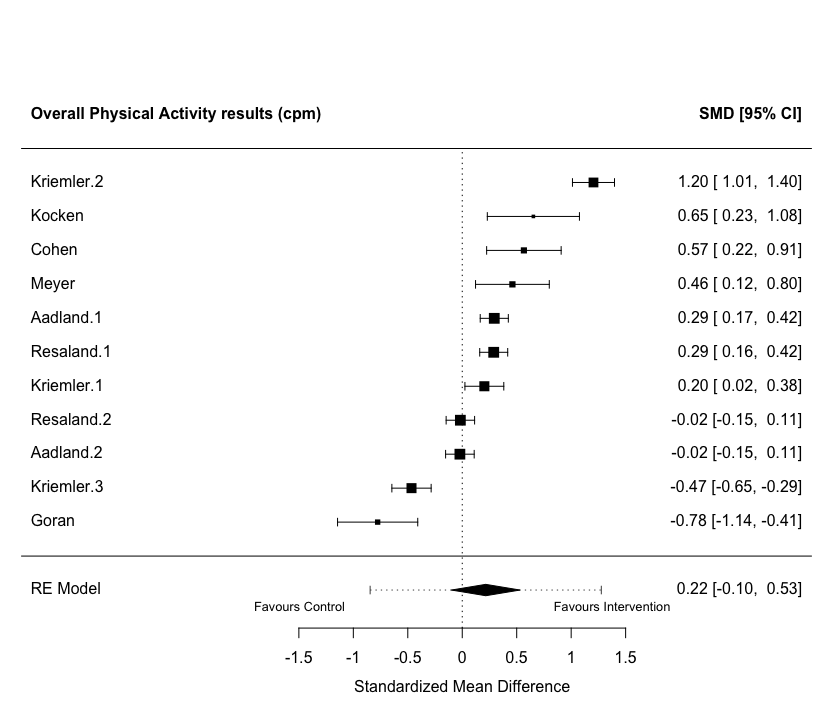


Each black square indicates an individual study’s SMD, with square size proportional to the study’s weight in the meta-analysis. Horizontal lines represent 95% CIs. The black diamond shape shows the overall effect estimate from the random-effects (RE) model, with the width indicating its 95% CI. The dotted vertical line indicates no effect (SMD = 0). Abbreviations: SMD, standardised mean difference; CI, confidence interval; RE model, Random Effects model.)

**MVPA (min/day) forest plot**

Each black square indicates an individual study’s SMD, with square size proportional to the study’s weight in the meta-analysis. Horizontal lines represent 95% CIs. The black diamond shape shows the overall effect estimate from the random-effects (RE) model, with the width indicating its 95% CI. The dotted vertical line indicates no effect (SMD = 0). Abbreviations: SMD, standardised mean difference; CI, confidence interval; RE model, Random Effects model.)

**
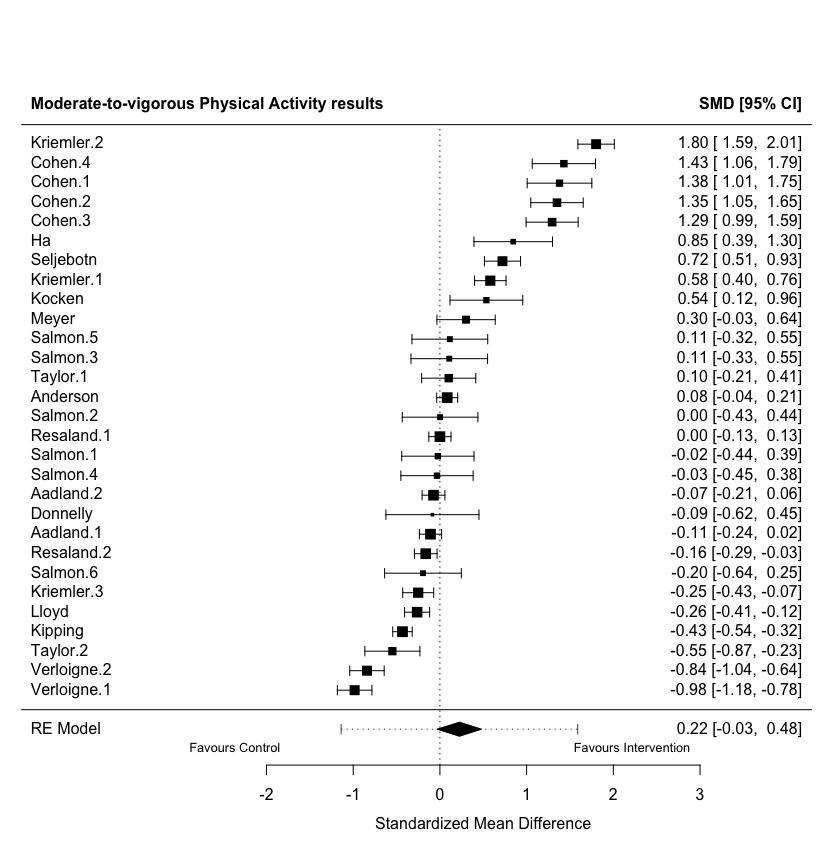
**

**LPA (min/day) forest plot**


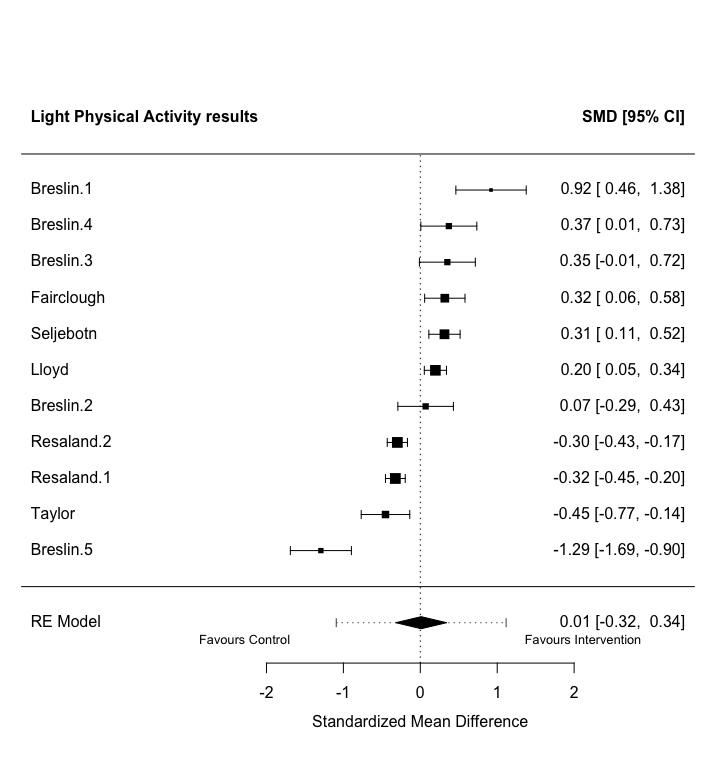


Each black square indicates an individual study’s SMD, with square size proportional to the study’s weight in the meta-analysis. Horizontal lines represent 95% CIs. The black diamond shape shows the overall effect estimate from the random-effects (RE) model, with the width indicating its 95% CI. The dotted vertical line indicates no effect (SMD = 0). Abbreviations: SMD, standardised mean difference; CI, confidence interval; RE model, Random Effects model.)

**MPA (min/day) forest plot**


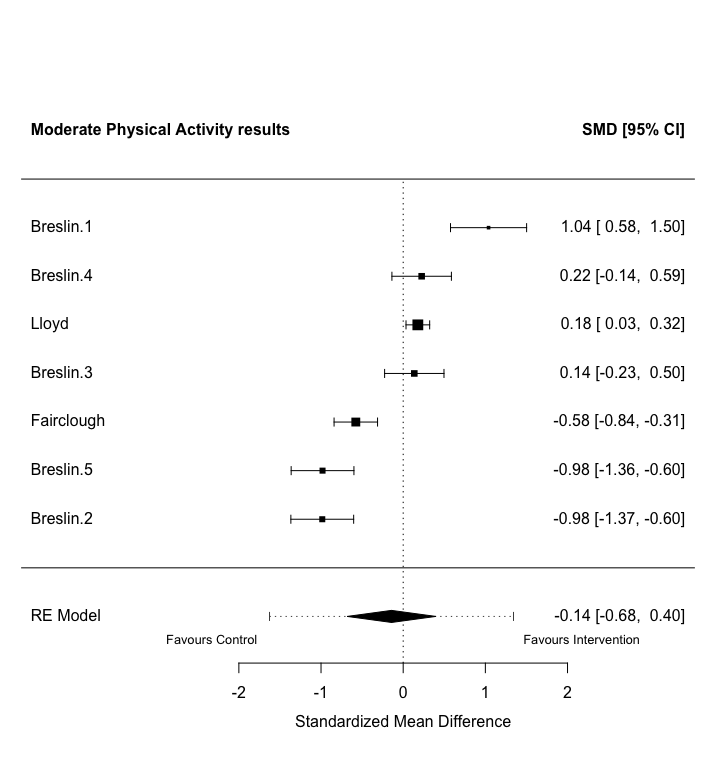


Each black square indicates an individual study’s SMD, with square size proportional to the study’s weight in the meta-analysis. Horizontal lines represent 95% CIs. The black diamond shape shows the overall effect estimate from the random-effects (RE) model, with the width indicating its 95% CI. The dotted vertical line indicates no effect (SMD = 0). Abbreviations: SMD, standardised mean difference; CI, confidence interval; RE model, Random Effects model.)

**VPA (min/day) forest plot**


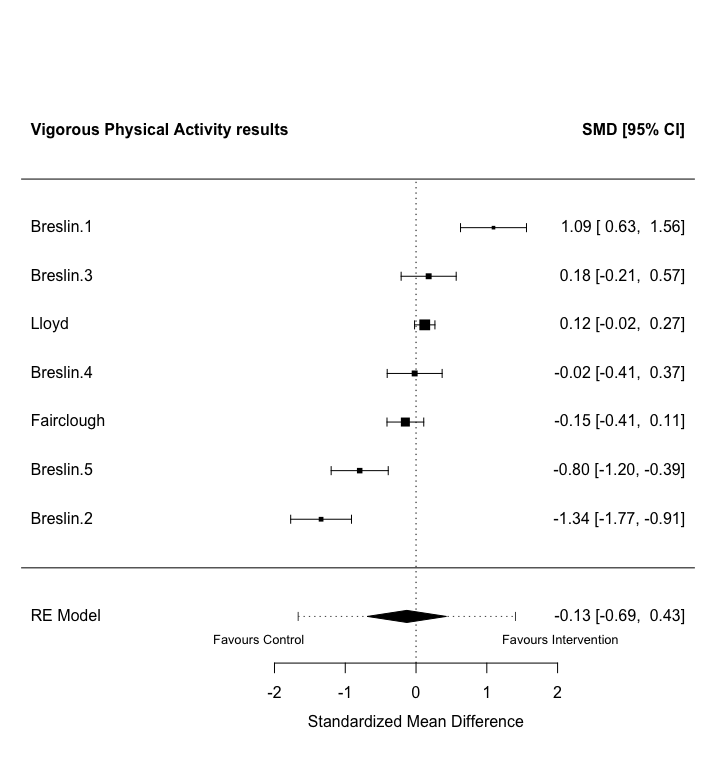


Each black square indicates an individual study’s SMD, with square size proportional to the study’s weight in the meta-analysis. Horizontal lines represent 95% CIs. The black diamond shape shows the overall effect estimate from the random-effects (RE) model, with the width indicating its 95% CI. The dotted vertical line indicates no effect (SMD = 0). Abbreviations: SMD, standardised mean difference; CI, confidence interval; RE model, Random Effects model.)
