# Supplementary material for: School-Based Homework Interventions for Improving 24-hour Movement Behaviours in Primary School Children: A Systematic Review and Meta-Analysis
Source: Sports Med Open. 2025 Aug 9;11:94. doi: 10.1186/s40798-025-00898-7 (PMC12335427; doi:10.1186/s40798-025-00898-7)
Supplement: Supplementary file 6 — Supplementary Material 6 [file 40798_2025_898_MOESM6_ESM.docx]

**School-based homework interventions for improving 24-hour movement behaviours in primary school children: A systematic review and meta-analysis.**

Sports Medicine – Open

April Forrest, ***Corresponding Author.***

University of the West of Scotland, School of Health and Life Sciences, Hamilton International Technology Park, Stephenson Place, Blantyre, Glasgow, G72 0LH, UK,

april.forrest@uws.ac.uk.

Dr Duncan Buchan.

University of the West of Scotland, School of Health and Life Sciences, Hamilton International Technology Park, Stephenson Place, Blantyre, Glasgow, G72 0LH, UK.

Professor Nicholas Sculthorpe.

University of the West of Scotland, School of Health and Life Sciences, Hamilton International Technology Park, Stephenson Place, Blantyre, Glasgow, G72 0LH, UK.

Dr Lawrence Hayes.

Lancaster Medical School, Faculty of Health & Medicine, Sir John Fisher Driver, Lancaster University, Lancaster, LA1 4AT, UK.

Dr Samantha Robinson.

University of the West of Scotland, School of Health and Life Sciences, Hamilton International Technology Park, Stephenson Place, Blantyre, Glasgow, G72 0LH, UK.

Eggers test for publication bias in funnel plots.

| **Behaviour Outcome** | **Coefficient** | ***P*** | **95% CI** |
| --- | --- | --- | --- |
| Sleep | -1.0043 | 0.3189 | -5.0825; 3.0739 |
| SB | -0.0493 | 0.2651 | -0.3446; 0.2461 |
| PA (cpm) | 0.1403 | 0.8343 | -0.6401; 0.9207 |
| PA (min/day) | -0.0499 | 0.6597 | -0.5090; 0.4092 |
| MVPA | -0.1048 | 0.2500 | -0.7212; 0.5115 |
| LPA | -0.1807 | 0.6635 | -1.1147; 0.7533 |
| MPA | -0.4105 | 0.7978 | -2.5507; 1.7298 |
| VPA | -0.0018 | 0.9023 | -2.1407; 2.1371 |

Abbreviations: SB, sedentary behaviour; PA, physical activity; cpm, counts per minute; MVPA, moderate-to-vigorous physical activity; LPA, light physical activity; MPA, moderate physical activity; VPA, vigorous physical activity; CI, confidence interval.
