# Supplementary material for: School-Based Homework Interventions for Improving 24-hour Movement Behaviours in Primary School Children: A Systematic Review and Meta-Analysis
Source: Sports Med Open. 2025 Aug 9;11:94. doi: 10.1186/s40798-025-00898-7 (PMC12335427; doi:10.1186/s40798-025-00898-7)
Supplement: Supplementary file 7 — Supplementary Material 7 [file 40798_2025_898_MOESM7_ESM.docx]

April Forrest

PhD Student,

Division of Sport, Exercise and Health,

School of Health and Life Sciences,

University of the West of Scotland,

Lanarkshire Campus

Hamilton International Technology Park

South Lanarkshire

G72 0LH

Scotland

Dear Editor,

Please find attached the revision of the manuscript entitled: **‘School-based homework interventions for improving 24-hour movement behaviours in primary school children: A systematic review and meta-analysis’** for re-consideration for publication in *Sports Medicine - Open.*

Please also find attached the responses to the comments in the rebuttal. We wish to thank all those involved in the initial review process and appreciate all of the comments. We hope our responses adequately address the comments made and the edits are sufficient for publication.

Yours Sincerely,

April Forrest, Dr Duncan Buchan, Prof. Nicholas Sculthorpe, Dr Lawrence Hayes, Dr Samantha Robinson.
